# Microbiota-based markers predictive of development of *Clostridioides difficile* infection

**DOI:** 10.1038/s41467-021-22302-0

**Published:** 2021-04-14

**Authors:** Matilda Berkell, Mohamed Mysara, Basil Britto Xavier, Cornelis H. van Werkhoven, Pieter Monsieurs, Christine Lammens, Annie Ducher, Maria J. G. T. Vehreschild, Herman Goossens, Jean de Gunzburg, Marc J. M. Bonten, Surbhi Malhotra-Kumar, the ANTICIPATE study group, Annemarie Engbers, Marieke de Regt, Lena M. Biehl, Oliver A. Cornely, Nathalie Jazmati, Marie-Noelle Bouverne, Frederique Sablier-Gallis, France Mentré, Uta Merle, Andreas Stallmach, Jan Rupp, Johannes Bogner, Christoph Lübbert, Gerda Silling, Oliver Witzke, Achilleas Gikas, Sofia Maraki, George Daikos, Sotirios Tsiodras, Athanasios Skoutelis, Helen Sambatakou, Miquel Pujol, M. Angeles Dominguez-Luzon, Jose M. Aguado, Emilio Bouza, Javier Cobo, Jesús Rodríguez-Baño, Benito Almirante, Julian de la Torre Cisneros, Simin A. Florescu, Maria Nica, Andrei Vata, Adriana Hristea, Mihaela Lupse, Delia Herghea, Deborah Postil, Olivier Barraud, Jean-Michel Molina, Victoire De Lastours, Thomas Guimard, Jean-Philippe Talarmin, Xavier Duval, Louis Bernard, Odile Launay

**Affiliations:** 1 Laboratory of Medical Microbiology, Vaccine & Infectious Disease Institute, University of Antwerp, Wilrijk, Belgium; 2 Microbiology Unit, Interdisciplinary Biosciences, Belgian Nuclear Research Centre, SCK–CEN, Mol, Belgium; 3 Julius Center for Health Sciences and Primary Care, University Medical Center Utrecht, Utrecht University, Utrecht, the Netherlands; 4 Da Volterra, Paris, France; 5 Department of Internal Medicine, Center for Integrated Oncology Aachen Bonn Cologne Duesseldorf, University of Cologne, Cologne, Germany; 6 German Centre for Infection Research (DZIF), partner site Bonn-Cologne, Cologne, Germany; 7 Department of Internal Medicine, Infectious Diseases, University Hospital Frankfurt, Goethe University Frankfurt, Frankfurt am Main, Germany; 8 Department of Medical Microbiology, University Medical Center Utrecht, Utrecht University, Utrecht, the Netherlands; 45 Present Address: Department of Biomedical Sciences, Institute of Tropical Medicine, Antwerp, Belgium; 9 Department of Internal Medicine, University Medical Center Utrecht, Utrecht, the Netherlands; 10 Institute for Medical Microbiology, Immunology and Hygiene, University of Cologne, Cologne, Germany; 11 INSERM, Paris, France; 12 Paris Diderot University, IAME, Paris, France; 13 Universitätsklinikum Heidelberg, Heidelberg, Germany; 14 Universitätsklinikum Jena, Jena, Germany; 15 Universitätsklinikum Schleswig-Holstein, Lübeck, Germany; 16 Klinikum der Universität München, München, Germany; 17 Universitätsklinikum Leipzig, Leipzig, Germany; 18 Universitätsklinikum Aachen, Aachen, Germany; 19 Universitätsklinikum Essen, Essen, Germany; 20 University Hospital of Heraklion, Heraklion, Greece; 21 Laiko General Hospital, Athens, Greece; 22 University General Hospital ATTIKON, Athens, Greece; 23 Evangelismos General Hospital, Athens, Greece; 24 Ippokratio Hospital, Athens, Greece; 25 Hospital Universitari de Bellvitge, Universitat de Barcelona, IDIBELL, Barcelona, Spain; 26 Hospital Universitario 12 de Octubre, Madrid, Spain; 27 Hospital Universitario Gregorio Marañón, Madrid, Spain; 28 Hospital Universitario Ramón y Cajal, Madrid, Spain; 29 Hospital Universitario Virgen Macarena, Sevilla, Spain; 30 Hospital Universitari Vall d’Hebrón, Barcelona, Spain; 31 IMIBIC-Hospital Universitario Reina Sofia, UCO, Cordoba, Spain; 32 Infectious and Tropical Diseases Hospital Dr. Victor Babes, Bucharest, Romania; 33 Clinical Hospital of Infectious Diseases of Iasi, Iasi, Romania; 34 The National Institute of Infectious Diseases Matei Bals, Bucharest, Romania; 35 Cluj Napoca Infectious disease Clinical Hospital, Cluj Napoca, Romania; 36 Oncology Institute Prof. Dr. I Chiricuta, Cluj Napoca, Romania; 37 Centre hospitalier universitaire Dupuytren, Limoges, France; 38 Hôpital St Louis, Paris, France; 39 APHP Beaujon, Paris, France; 40 Centre Hospitalier Départemental Vendée, La Roche sur Yon, France; 41 CH de Cornouaille, Quimper, France; 42 APHP Bichat, Paris, France; 43 Centre hospitalo-universitaire de Tours, Tours, France; 44 APHP Hôpital Cochin, Paris, France

**Keywords:** Next-generation sequencing, Microbiome, Predictive markers

## Abstract

Antibiotic-induced modulation of the intestinal microbiota can lead to *Clostridioides difficile* infection (CDI), which is associated with considerable morbidity, mortality, and healthcare-costs globally. Therefore, identification of markers predictive of CDI could substantially contribute to guiding therapy and decreasing the infection burden. Here, we analyze the intestinal microbiota of hospitalized patients at increased CDI risk in a prospective, 90-day cohort-study before and after antibiotic treatment and at diarrhea onset. We show that patients developing CDI already exhibit significantly lower diversity before antibiotic treatment and a distinct microbiota enriched in *Enterococcus* and depleted of *Ruminococcus*, *Blautia, Prevotella* and *Bifidobacterium* compared to non-CDI patients. We find that antibiotic treatment-induced dysbiosis is class-specific with beta-lactams further increasing enterococcal abundance. Our findings, validated in an independent prospective patient cohort developing CDI, can be exploited to enrich for high-risk patients in prospective clinical trials, and to develop predictive microbiota-based diagnostics for management of patients at risk for CDI.

## Abstract (web summary)

*Clostridioides difficile* infection (CDI) is the most common cause of antibiotic-associated diarrhoea (AAD); however, markers predictive of CDI or AAD development are as yet lacking. Here, to identify markers predictive of CDI, the authors profile the intestinal microbiota of 945 hospitalised patients from 34 hospitals in 6 different European countries and show distinct microbiota enriched in *Enterococcus* and depleted of *Ruminococcus, Blautia, Prevotella* and *Bifidobacterium* compared to non-CDI patients.

## Introduction

*Clostridioides difficile* is the most common cause of infectious antibiotic-associated diarrhea (AAD) and is the pathogen responsible for the largest number of healthcare-associated infections world-wide^1–3^. *C. difficile* infection (CDI) is characterized by watery stool accompanied by toxin-mediated inflammation of the bowel where primary risk factors include hospitalization, age, colonization by toxigenic *C. difficile*, and most importantly, antibiotic exposure where use of fluoroquinolones (FQNs), clindamycin, carbapenems, cephalosporins, and penicillins combined with beta-lactamase inhibitors (PBLs) are associated with increased CDI risk^4–9^. However, markers predictive of CDI or AAD development are as yet lacking. Such markers could be utilized to stratify patients into different risk categories and to enrich patient populations for clinical trials assessing preventive measures against or therapeutics for CDI.

Patients suffering from CDI, as well as from other forms of AAD harbor a disrupted intestinal microbiota characterized by reduced diversity and elevated levels of *Enterococcus* alongside reduced levels of members of the Bacteroidetes phylum, the Lachnospiraceae and Ruminococcaceae families, and *Prevotella* spp. during disease manifestation^10–20^. 16S rRNA gene profiling provides a useful method for studying changes in microbial composition. More importantly, it might allow identification of microbial markers predictive of the risk of CDI development. Although the microbial composition at CDI onset has been well-studied, the pre-CDI microbiota remains largely unexplored. To our knowledge, only one prospective study has investigated microbial composition as a potential predictor of CDI^18,21^. This single-center Canadian study^18^, part of a larger clinical study assessing CDI risk factors^21^, demonstrated that the absence or reduction in Clostridiales, namely members of Clostridiales Incertae Sedis XI, in the intestinal microbiota was associated with an increased risk of CDI^18^. Collateral damage on the microbiota induced by antibiotic treatment has been further shown to result in reduced alpha diversity, as well as an increase in the presence of antibiotic resistance genes, and long-lasting effects ranging from weeks to years depending on the antibiotic^22–25^. Several small studies in healthy adults have investigated specific short-term changes induced by antibiotics, often in combination with multiple compounds^25–28^. None of these, however, link antibiotic-induced dysbiosis to development of CDI.

In this multi-center, observational, prospective study, we investigated the intestinal microbiota of hospitalized patients aged 50 years and above in 34 hospitals across six European countries prior to antibiotic therapy with the aim of identifying robust microbial markers predictive of CDI and AAD development utilizing 16S rRNA gene profiling combined with a high-resolution sequence typing approach. Furthermore, longitudinal perturbations induced by different antibiotic classes were studied in relation to the CDI-specific and AAD-specific microbiota. Here we show that patients developing CDI exhibit significantly lower microbial diversity prior to antibiotic treatment and a distinct microbiota enriched in *Enterococcus* and depleted of *Ruminococcus*, *Blautia, Prevotella*, and *Bifidobacterium* spp. compared to non-CDI patients. By validating our findings in an independent prospective cohort, we show that these microbial markers are applicable in a geographically diverse patient population. Further, we show that broad-spectrum antibiotic treatment induces class-specific dysbiosis with beta-lactam antibiotics particularly increasing enterococcal abundance. Collectively, our findings can be exploited to enrich for patients at high risk of CDI development in prospective clinical trials and to develop predictive microbiota-based diagnostics for management of patients at risk for CDI.

## Results

### Study population and design

**Fig. 1:**
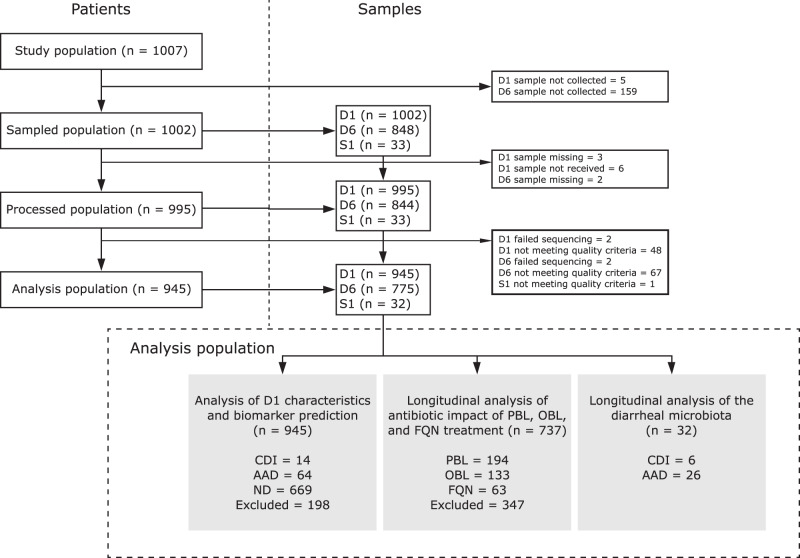
Patient and sample flow in the study. The flow chart provides an overview of participating patients in each processing step, number of samples collected at each timepoint, and reasons for sample exclusion or non-collection. D1: rectal swab sample collected upon study enrollment. D6: rectal swab collected ~6 days after initiation and at the end of antibiotic treatment. S1: stool sample collected at the first occurrence of diarrhea (variable time-point). AAD: patients with non-*C. difficile* antibiotic-associated diarrhea. CDI: patients with confirmed *C. difficile* infection. ND: non-diarrheic patients. PBL: penicillin + beta-lactamase inhibitor. OBL: other beta-lactamase antibiotics. FQN: fluoroquinolones.

The ANTICIPATE study recruited 1007 patients of which 1002 provided rectal swabs at D1 and 848 at D6 (Fig. 1). Of the 135 patients who developed diarrhea during a period of 90 days following study inclusion, 15 were diagnosed with CDI, as described in van Werkhoven et al.^29^ Stool samples from 33 of the 135 patients with diarrhea, including 6 patients diagnosed with CDI, collected at the occurrence of the first diarrheal episode (S1) were available for analysis (Fig. 1, Table 1). Following 16S rRNA gene sequencing, 945 and 775 rectal swab samples collected at D1 and D6, respectively, and S1 samples from 32 diarrheic patients (including stools from 6 CDI patients) passed data pre-processing and quality filtering criteria and were analyzed further. Demographics of the 945 patients included in this study are detailed in Table 1. In the studied population, 390 patients received antibiotic treatment with drugs belonging to one of the following classes of broad-spectrum antibiotics: PBLs, other beta-lactam antibiotics (OBLs), and FQNs (Table 1).

**Table 1:** Patient demographics of the analyzed study population.

*Total number of patients*	*945*
Age (median [IQR])	70 [61–79]
Male gender (%)	557 (58.9)
Myocardial infarction (%)	78 (8.25)
Congestive heart failure (%)	134 (14.2)
Peripheral vascular disease (%)	143 (15.1)
Cerebrovascular disease (%)	81 (8.57)
COPD (%)	144 (15.2)
Connective tissue disease (%)	53 (5.61)
Peptic ulcer disease (%)	50 (5.29)
Diabetes mellitus (%)	268 (28.4)
Moderate to severe chronic kidney disease (%)	127 (13.4)
Hemiplegia (%)	16 (1.69)
Leukemia (%)	55 (5.82)
Malignant lymphoma (%)	68 (7.20)
Solid tumor (%)	208 (22.0)
Liver disease (%)	88 (9.31)
AIDS (%)	10 (1.06)
Intestinal obstruction (%)	5 (0.53)
Inflammatory bowel disease (%)	14 (1.48)
Other non-specified comorbidities (%)	538 (56.9)
Has history of CDI (%)	14 (1.48)
Developed CDI within study period (%)	14 (1.48)
Developed AAD within study period (%)	64 (6.77)

*Country of origin*	
France (%)	210 (22.2)
Germany (%)	145 (15.3)
Greece (%)	85 (8.99)
The Netherlands (%)	14 (1.48)
Romania (%)	184 (19.5)
Spain (%)	307 (32.2)

*Antibiotic treatment received from D1 to D5*	
Penicillin + beta-lactamase inhibitor (PBL, %)	194 (20.5)
Other beta-lactam antibiotics (OBL, %)	133 (14.1)
Fluoroquinolones (FQN, %)	63 (6.67)
Combination therapy and other (%)	347 (36.7)
Patients without a D6 sample (%)	208 (22.0)

*IQR* interquartile range, *COPD* chronic obstructive pulmonary disease, *AIDS* acquired immune deficiency syndrome, *AAD* patients with non-*C. difficile* antibiotic-associated diarrhea, *CDI* patients with confirmed *C. difficile* infection, *D1* rectal swab sample collected upon study enrollment, *D6* rectal swab collected ~6 days after initiation and at the end of antibiotic treatment.

Influence of baseline characteristics—gender, country of origin, and age by decades—on the fecal microbiome of the 945 patients was assessed at D1 (Supplementary Table 1). Certain differences in both alpha and beta diversity were observed in the microbiota, namely when patients were stratified by countries of origin (Supplementary Results, Supplementary Table 1, and Supplementary Fig. 1). However, the aim of this study was to identify biomarkers predictive of CDI that were robust enough to transcend country, diet, and gender differences in the intestinal microbiota. Therefore, patients were not stratified by baseline characteristics.

### High-resolution 16S rRNA profiling defines *C. difficile* prevalence

Upon OTU clustering using a 97% cut-off, a single OTU classified as *Clostridium XI* was identified in 556 of the 945 (58.8%) patient samples at D1. High-resolution analysis of this OTU by oligotyping revealed two distinct types of raw reads, one corresponding to *C. difficile* and the other to *Clostridium bartlettii* (also known as *Intestenibacter bartlettii*^30^), a known anaerobic occupant of the human intestine^31^ (Supplementary Results).

**Fig. 2:**
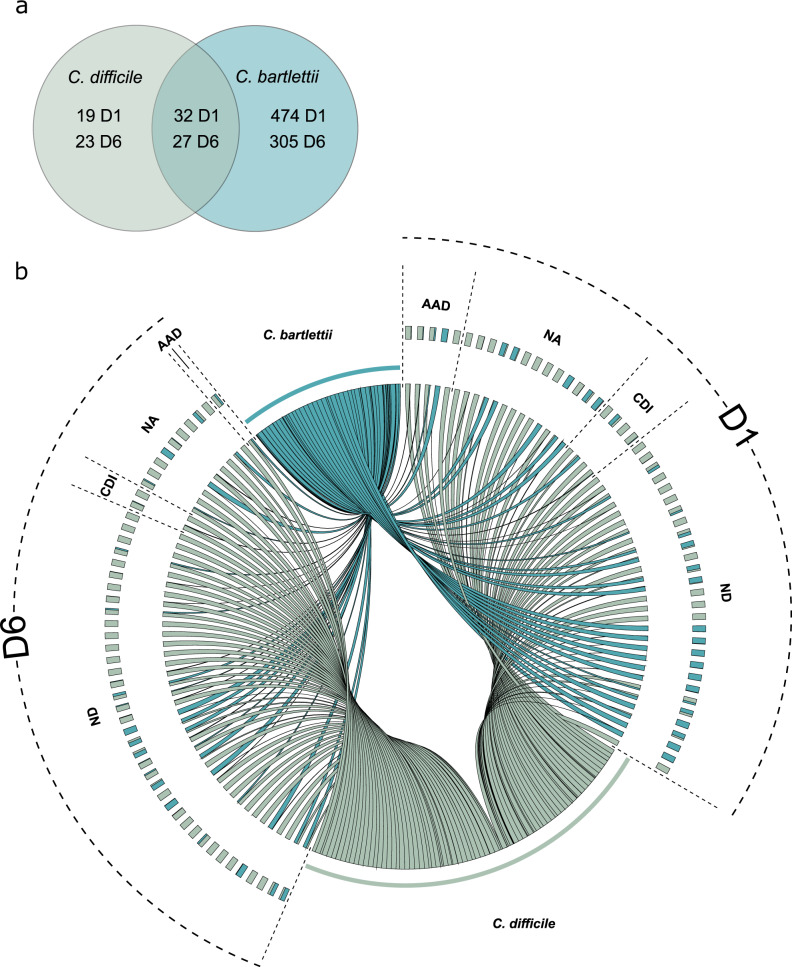
*C. difficile* and *C. bartlettii* carriage in the analyzed population at D1 and D6. Oligotyping revealed diversity within the OTU classified as *Clostridium XI* wherein reads were divided into *C. difficile* (green) and *C. bartlettii* (blue). **a** Rectal swab samples collected at the time of study enrollment (D1), and ~6 days later at the end of treatment (D6) harbored reads classified as both *C. difficile* and *C. bartlettii*. Patients that were defined as *C. difficile* carriers due to the presence of *C. difficile* reads after oligotyping showed no clear link to clinical outcome. **b** Similar carriage rates of the OTU *Clostridium XI* were identified in patients both at D1 (5.40%, *n* = 51) and D6 (6.78%, *n* = 50) with varying relative abundances of *C. difficile* and *C. bartlettii* reads as demonstrated in the Circos plot. Patients at D6 generally show higher relative abundance of *C. difficile* than of *C. bartlettii* compared to D1. D1: rectal swab sample collected upon study enrollment. D6: rectal swab collected ~6 days after initiation and at the end of antibiotic treatment. AAD: patients with non-*C. difficile* antibiotic-associated diarrhea. CDI: patients with confirmed *C. difficile* infection. ND: non-diarrheic patients. NA: patients without known CDI status and/or early study termination. OTU: operational taxonomic unit.

Oligotyping identified *C. difficile* in 51 patients (5.40%) at D1 with an average relative abundance of 0.82% (SD = 2.67, Fig. 2a). Of these, four patients developed CDI within 1–3 days of the D1 sampling (i.e., study inclusion). Relative abundance of *C. difficile* at D1 was slightly higher in the four CDI patients (1.72%, SD = 2.18) than in the 47 patients who did not develop CDI within the 90-day study period (0.74%, SD = 2.70). All four CDI patients underwent treatment with metronidazole and/or vancomycin between the D1 and D6 sampling time-points and in two patients, *C. difficile* was not detected at D6.

Of the 945 studied patients, 737 provided both D1 and D6 samples. *C. difficile* was detected in 50 patients at D6 (6.78%) at a 2.5-fold higher average relative abundance (2.02%, SD = 6.14) compared to D1 (Fig. 2b). Only 13 patients carried *C. difficile* at both sampling time-points. *C. difficile* was detected in all six analyzed stools originating from patients with confirmed CDI at an average relative abundance of 0.26% (SD = 0.29), and in two stools from AAD patients that tested negative for CDI with a relative abundance of 0.01% and 0.02%.

### Distinct microbial markers predictive of CDI and AAD development

**Fig. 3:**
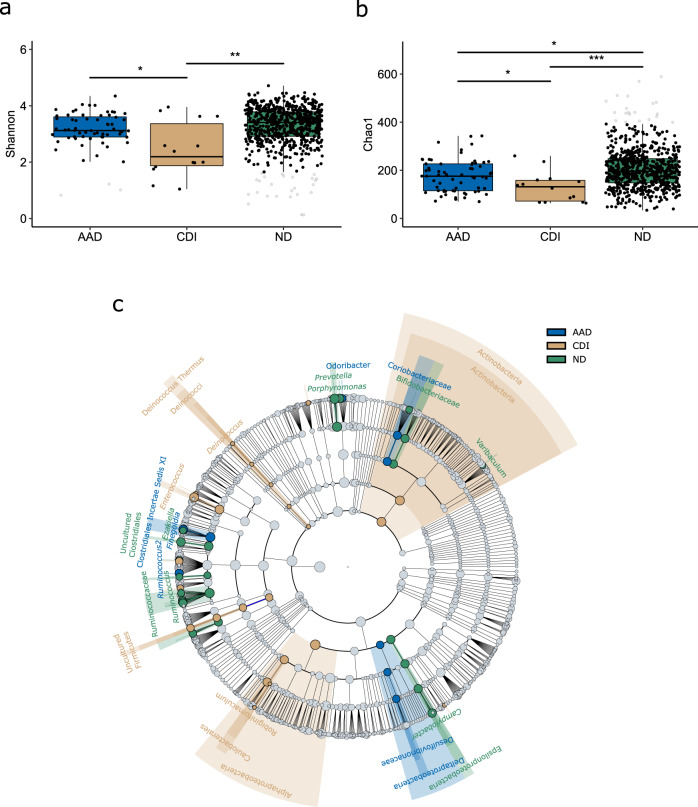
Characterization of microbial diversity in baseline (D1) samples. **a** CDI patients (*n* = 14, brown) display distinctly lower alpha diversity expressed by the Shannon index compared to AAD (*n* = 64, blue, *p* = 0.037) and ND patients (*n* = 669, green, *p* = 0.005) at D1. AAD patients similarly display lower Shannon diversity compared to ND patients, however not sufficient to be statistically significant (*p* = 0.087). **b** Both CDI and AAD patients display lower diversity expressed by the Chao1 index compared to ND patients (*p* = 0.001 and 0.017, respectively) at D1. Compared to patients who develop AAD, CDI patients display lower Chao1 indices (*p* = 0.049). **c** Cladogram generated by LEfSe demonstrating significantly higher abundances of Actinobacteria, Alphaproteobacteria, and *Enterococcus* spp. in the gut microbiota of CDI patients at baseline (D1) compared to AAD and ND patients. The cladogram shows distinctly abundant taxa of interest. For more details, see Supplementary Fig. 4. Alpha diversity indices were compared using the non-parametric two-sided Wilcoxon rank sum test followed by Bonferroni correction of *p*-values. Box plots indicate median (middle line), 25th, 75th percentile (box), and 5th and 95th percentile (whiskers) as well as outliers (gray single dots). AAD: patients with non-*C. difficile* antibiotic-associated diarrhea. CDI: patients with confirmed *C. difficile* infection. ND: non-diarrheic patients. LEfSe: Linear discriminant analysis effect size. LDA: linear discriminant analysis score. *p < 0.05. **p ≤ 0.01. ***p ≤ 0.001.

Alpha diversity at D1 was lower in patients developing CDI, compared to those developing AAD or ND (Fig. 3a, b, *p* ≤ 0.049). AAD patients also had lower Chao1 indices compared to ND patients whereas a decreasing trend was observed in Shannon indices, however, not sufficient to be significant (Fig. 3a, b, *p* = 0.017 and *p* = 0.087, respectively). Furthermore, at D1, the microbiota composition differed between patients developing CDI and the other two groups (Fig. 3c, *p* ≤ 0.025), and between AAD patients and ND patients (*p* = 0.002), with the most pronounced differences observed between CDI and ND patients (*p* < 0.001).

Microbial composition at D1 was compared between the three patient groups (CDI, AAD, and ND) for identification of predictive microbial markers. Patients who developed CDI harbored elevated levels of the pathobiont *Enterococcus* (Fig. 3c, Table 2, and Supplementary Fig. 4). AAD patients harbored elevated levels of the family Clostridiales Incertae Sedis XI, *Ruminococcus* (*R. torques, R. faecis, R. lactaris*, and *Clostridium glycyrrhizinilyticum*), and *Blautia* spp. (*B. wexlerae*, *B. obeum*, and *B. faecis*, Supplementary Tables 2–6), whereas ND patients primarily exhibited a higher abundance of *Blautia luti*, *Prevotella*, uncultured Clostridiales, *Porphyromonas* (*P. bennonis*, *P. asaccharolytica*, *P. uenonis*), *Campylobacter*, and *Ezakiella*, in addition to members of the Bifidobacteriales family. When comparing CDI versus non-CDI (including AAD, ND, and non-CDI tested diarrheic cases) patients at D1, several OTUs showed lower abundance among CDI patients, which included six *Bifidobacterium* spp. and three *Blautia* spp. *B. wexlerae*, *B. obeum*, and *B. faecis* (Supplementary Tables 2, 3, and 6).

**Table 2:** Identification of microbiota-based markers at D1.

Group	OTU	LDA	*p-*value	Genus
AAD	Otu9	4.25	0.0423	*Finegoldia*
	Otu30	3.85	0.0280	*Blautia*
	Otu31	3.66	0.0207	*Ruminococcus2*
	Otu106	3.32	0.0131	Uncultured Clostridiales
	Otu165	2.66	0.0402	Uncultured Lachnospiraceae
	Otu300	2.81	0.0054	*Odoribacter*
CDI	Otu1	4.29	0.0002	*Enterococcus*
	Otu143	3.75	0.0421	*Phenylobacterium*
	Otu163	3.28	0.0455	*Dorea*
	Otu359	2.87	0.0169	*Oscillibacter*
ND	Otu56	3.79	0.0048	*Porphyromonas*
	Otu69	3.59	0.0047	*Porphyromonas*
	Otu648	3.18	0.0035	*Blautia*
	Otu2540	3.37	0.0401	*Ruminococcus*
	Otu127	3.33	0.0001	*Oscillibacter*
	Otu137	3.11	0.0354	Uncultured Lachnospiraceae
	Otu1026	2.86	0.0277	Uncultured Lachnospiraceae
	Otu974	3.06	0.0003	*Roseburia*
	Otu262	2.58	0.0055	*Butyricoccus*
	Otu294	2.85	0.0309	Uncultured Lachnospiraceae
	Otu399	2.94	0.0006	Uncultured Ruminococcaceae

The microbiota of patients developing CDI (*n* = 14) and AAD (*n* = 64), and ND patients (*n* = 699) was compared to identify predictive biomarkers at D1. Distinctly abundant OTUs associated with each condition were identified using linear discriminant analysis effect size (LEfSe, LDA > 2.0). AAD: patients with non-*C. difficile* antibiotic-associated diarrhea. For more details, see Fig. 3 and Supplementary Fig. 4. CDI: patients with confirmed *C. difficile* infection, ND: non-diarrheic patients, D1: rectal swab sample collected upon study enrollment, LDA: linear discriminant analysis score, OTU: Operational taxonomic unit.

The OTU with the strongest association at D1 with the subsequent development of CDI, classified as *Enterococcus*, consisted of two types of raw reads distinguished by seven nucleotides (constituting a 98.4% sequence homology). One was classified as one or multiple species of the group consisting of *E. hirae*, *E. villorum*, *E. ratti*, *E. faecium*, or *E. durans* (oligotype 1, 63.5% of all OTU reads) and the other as *E. faecalis* (oligotype 2, 36.5% of all OTU reads) both with 100% sequence identity and length.

In order to further elucidate the specific species constituting oligotype 1, we performed shotgun metagenomic sequencing on D1 samples from patients developing CDI. Oligotype 1 was primarily constituted by *E. faecium* and *E. villorum* corresponding to average relative abundances of 57% and 32%, respectively, within the oligotype (Supplementary Table 7). Similarly D1 samples from non-CDI patients underwent shotgun sequencing and two of the most prevalent OTUs classified as *Bifidobacterium* spp. and *Blautia* spp. were delineated to be *Bifidobacterium adolescentis*, *B. catenulatum*, *B. dentium*, and *Blautia wexlerae* and *B. obeum*, respectively (Supplementary Table 7). OTUs classified as *Ruminococcus* and *Alistipes* were also abundant in non-CDI patients and were further speciated as, *R. torques* and *R. lactaris* as the most prevalent of the oligotypes together with *R. bromii*, and *A. onderdonkii* and *A. finegoldia*, respectively.

### Validation of microbial markers predictive of CDI

To verify the generalizability and potential of the identified microbiota-based biomarkers predictive of CDI, we analyzed a previously published dataset^18^. This dataset, generated from a Canadian cohort of elderly patients who developed CDI with matched non-diarrheic patient controls served as an independent validation of the microbial markers identified in this study to be predictive of CDI. Briefly, prospective fecal samples had been collected from patients developing CDI (*n* = 25) as well as from age-matched and gender-matched non-diarrheic control patients (*n* = 25), and resulted in 24 and 25 baseline samples from patients developing CDI and from non-CDI controls, respectively, after processing. In concordance with our results, baseline samples from patients who subsequently developed CDI (median time to development, 5 days) in the validation dataset also harbored elevated levels of a single enterococcal OTU (Table 3). Also, in concordance with our data, the OTU classified as *Enterococcus* spp. corresponded to two oligotypes, one classified as *E. faecalis* (73.81% overall relative abundance) and the other as *E. faecium*, *E. hirae*, *E. villorum*, *E. ratti*, or *E. durans* (26.19% overall relative abundance, Supplementary Table 7). It was further noted that 91.7% (*n* = 22) of the patients who proceeded to develop CDI within this dataset (*n* = 24) were colonized by *C. difficile* (OTU classified as *Clostridium XI*) upon study enrollment. Patients who did not develop CDI in the validation dataset harbored elevated levels of *Ezakiella*, *Odoribacter*, and *Ruminococcus* spp. together with uncultured Clostridiales (Table 3). These also corresponded to the species identified by oligotyping in our data (Supplementary Tables 6 and 8).

**Table 3:** Validation of identified microbiota-based markers predictive of CDI.

OTU	OTU classification	ANTICIPATE dataset				Validation dataset			
		Patient group	Average relative abundance (%)	LDA	*p*-value	Patient group	Average relative abundance (%)	LDA	*p*-value
Otu1	*Enterococcus*	CDI	1.86	4.27	0.0001	CDI	6.40	4.41	0.0037
Otu9*	*Finegoldia*	CDI	4.18	4.32	0.0200	Non-CDI	2.76	3.95	0.0009
Otu31	*Ruminococcus2*	Non-CDI	1.32	3.53	0.0059	Non-CDI	2.04	3.70	0.0302
Otu64	*Ezakiella*	Non-CDI	0.77	3.59	0.0320	Non-CDI	1.17	3.79	0.0190
Otu106	Uncultured Clostridiales	Non-CDI	0.34	3.18	0.0117	Non-CDI	1.09	4.06	0.0006
Otu300	*Odoribacter*	Non-CDI	0.20	2.79	0.0496	Non-CDI	0.39	3.64	0.0022

Microbiota-based biomarkers identified in the main study were validated by conducting biomarker identification in an independent patient cohort^18^. Potential biomarkers associated with CDI (*n* = 24) and non-CDI (*n* = 25) patients were identified in the validation cohort. Distinctly abundant OTUs associated with each condition were identified using linear discriminant analysis effect size (LEfSe, LDA > 2.0). OTU9(*) classified as *Finegoldia* was associated with patients developing CDI (*n* = 14) when comparing with non-CDI patients (*n* = 733) in the main study. The non-CDI group in our study is constituted by (non-CDI) AAD as well as ND patients, and further investigation (Supplementary Tables 3 and 5) revealed this OTU to be strongly associated with patients developing AAD at D1. AAD: patients with non-*C. difficile* antibiotic-associated diarrhea, CDI: patients with confirmed *C. difficile* infection, ND: non-diarrheic patients, Non-CDI: patients with non-*C. difficile* diarrhea or non-diarrheic patients, D1: rectal swab sample collected upon study enrollment, LDA: linear discriminant analysis score, OTU: operational taxonomic unit.

In addition, several taxa were also unique in the two datasets, especially those associated with non-CDI patients in our study were not identified in the validation dataset, and vice versa (Supplementary Table 9). These differences are likely due to variable sequencing technologies and depths in either study (15,000 reads in this study compared to 2000 in the validation dataset^18^). Finally, one OTU classified as *Finegoldia* spp. was associated with non-CDI patients in the validation dataset whereas our study revealed it to be linked to patients developing CDI when compared with non-CDI patients (Table 3). However, the non-CDI group in our study is constituted by (non-CDI) AAD as well as ND patients. Further assessment of taxa linked to CDI and AAD in our study (Supplementary Table 5) revealed *Finegoldia* spp. to be primarily associated with patients developing AAD rather than CDI, thereby correlating with the findings in the validation dataset.

### Antibiotic treatment induces class-specific microbial dysbiosis

**Fig. 4:**
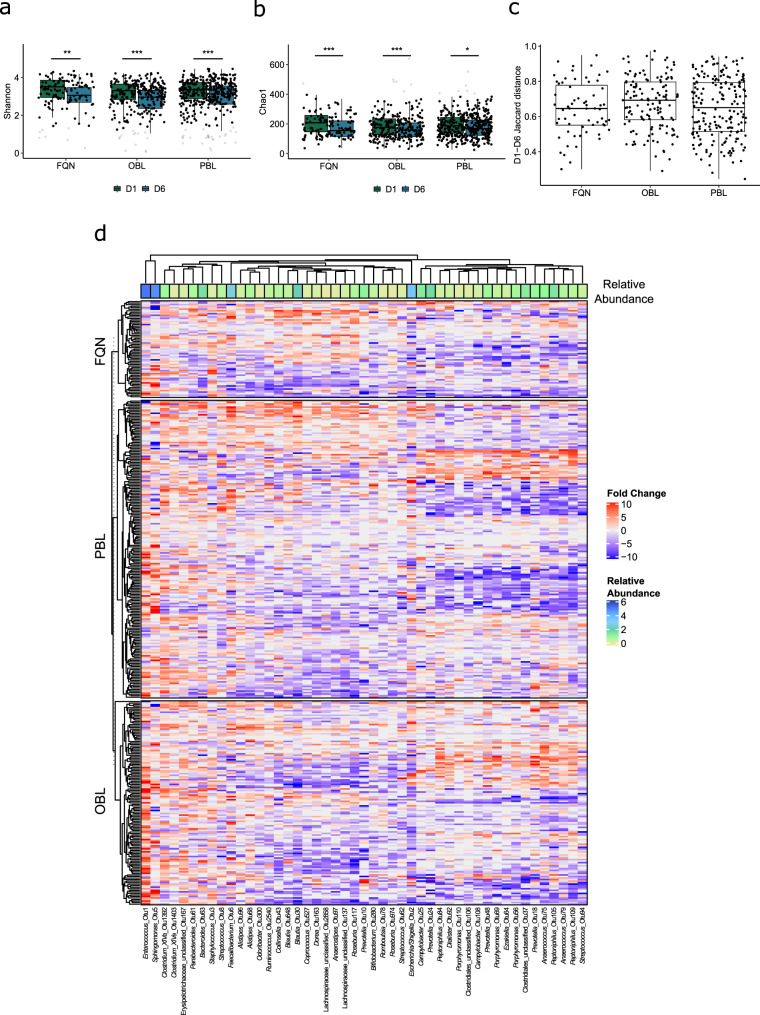
Longitudinal analysis of the impact of antibiotic therapy on the intestinal microbiota. Microbial diversity was compared prior to broad-spectrum antibiotic treatment at D1 (green) and after treatment at D6 (purple) following treatment with PBLs (*n* = 194), OBLs (*n* = 133), and FQNs (*n* = 63). **a** A distinct reduction was observed in Shannon diversity in patients treated with all antibiotic classes (*p* = 8.98*10^−6^, *p* = 2.06*10^−5^, and *p* = 0.007, respectively). **b** Similarly, distinct reductions in Chao1 diversity was observed following treatment with all antibiotic classes (*p* = 0.011, *p* = 0.001, and *p* = 9.26 × 10^−5^, respectively). **c** Treatment with each antibiotic class resulted in a shift in microbial composition illustrated by the Jaccard distances between the D1 and D6 samples. **d** The heatmap illustrates distinctly abundant taxa (LDA > 3.0) identified using LEfSe. For more details, see Supplementary Table 10. Alpha diversity indices were compared using the paired two-sided non-parametric Wilcoxon signed rank test. Box plots in indicate median (middle line), 25th, 75th percentile (box), and 5th and 95th percentile (whiskers) as well as outliers (gray single dots). PBL: penicillin + beta-lactamase inhibitor. OBL: other beta-lactam antibiotics. FQN: fluoroquinolones. LDA: Linear discriminant analysis score. D1: rectal swab sample collected upon study enrollment. D6: rectal swab collected ~6 days after initiation and at the end of antibiotic treatment. LEfSe: linear discriminant analysis effect size. LDA: linear discriminant analysis score. OTU: operational taxonomic unit. *p < 0.05, **p ≤ 0.01, ***p ≤ 0.001.

Samples from 390 patients were available for longitudinal analysis of class-specific antibiotic-induced intestinal dysbiosis. Of these, 194, 133, and 63 patients had received treatment with PBL, OBL, and FQN, respectively, between the D1 and D6 sampling time-points (Table 1). Comparison of D1 and D6 samples from the 390 patients showed that treatment with all three antibiotic classes induced a decrease in alpha diversity together with a shift in beta diversity of the intestinal microbiota (*p* < 0.001 for both). Further investigation into specific changes induced by the individual antibiotic classes (PBL, OBL, and FQN) revealed a decrease in both Shannon (*p* ≤ 0.007, Fig. 4a) and Chao1 diversity indices (*p* ≤ 0.011, Fig. 4b) at D6, together with a change in microbiota composition (*p* < 0.001). Comparison of beta diversity distances between D1 and D6 samples within each antibiotic class showed that patients treated with OBLs exhibited the largest alterations in microbial composition compared to those receiving PBLs and FQNs (Fig. 4c). Next, we compared the microbial compositional differences between the D1 and D6 samples within each of the three antibiotic classes. Treatment with all classes resulted in alterations within the four most dominant phyla of the intestinal microbiota; Firmicutes, Bacteroidetes, Actinobacteria, and Proteobacteria (Fig. 4d, Supplementary Table 10). The majority of these observed changes affected members of the Clostridia class. Treatment with beta-lactams (PBL and OBL) primarily affected members of the Firmicutes phylum where Clostridiales (primarily Lachnospiraceae members, especially after OBL treatment), and Lactobacillales were the major affected orders. FQN treatment resulted in alterations within the same taxa as beta-lactam treatment, as well as others such as the class Bacteroidia, where the Porphyromonadaceae and Prevotellaceae families were most affected.

Although each antibiotic class resulted in its own unique dysbiotic microbiota after treatment, some of the observed changes were also common (Fig. 4d, Supplementary Table 10). All three classes reduced *Streptococcus* spp. and increased relative abundances of *Sphingomonas* spp. after treatment (Supplementary Results). Beta-lactam treatment (PBL and OBL) resulted in a remarkable increase in *Enterococcus* and *Clostridium* XIVa spp. Additionally, PBL and FQN treatment both resulted in reductions in uncultured Clostridiales, *Anaerococcus* and *Peptoniphilus* spp., as well as in *Porphyromonas* and *Prevotella* spp., both members of the Bacteroidales order, and in *Campylobacter* spp. Similarly, OBL and FQN treatment both resulted in a reduction in *Escherichia*/*Shigella* spp. However, some classes resulted in an opposing effect of the affected taxa; OBL treatment reduced *Staphylococcus* spp., whereas the same taxon was found to increase after FQN treatment. Similarly, PBL treatment reduced *Dialister* spp., whereas FQN treatment resulted in an increase. Additionally, OBL treatment resulted in a reduction of *Blautia* spp. among others.

Comparison of the D6 samples of patients receiving any of the three antibiotic classes showed dysbiotic profiles that were class-specific (*p* < 0.001 overall, and ≤0.006 for the pairwise comparisons). Patients treated with OBLs harbored the highest levels of *Enterococcus* spp. at D6 together with elevated levels of *Prevotella* spp., whereas PBL-treated patients harbored elevated levels of *Escherichia*/*Shigella* compared to patients treated with other antibiotics (Table 4). Similar to OBL-treated patients, patients receiving PBLs also harbored increased levels of *Prevotella* spp. at D6, but distinct members of this genus compared to those observed after OBL treatment. FQN treatment resulted in a higher relative abundance of members of the Lachnospiraceae and Ruminococcaceae families as well as *Blautia* spp. (Table 4).

**Table 4:** Characterization of class-specific differences in microbial composition after broad-spectrum antibiotic treatment.

Antibiotic class	OTU	LDA	*p*-value	Genus
PBL	Otu24	3.97	0.002	*Prevotella*
	Otu2	3.85	0.000	*Escherichia/Shigella*
	Otu1392	3.48	0.004	*Clostridium_XlVa*
	Otu56	3.14	0.019	*Porphyromonas*
OBL	Otu1	4.38	0.000	*Enterococcus*
	Otu21	3.92	0.018	Uncultured Lachnospiraceae
	Otu18	3.83	0.004	*Prevotella*
	Otu48	3.79	0.000	*Prevotella*
	Otu68	3.63	0.045	*Alistipes*
	Otu167	3.51	0.000	Uncultured Erysipelotrichaceae
FQN	Otu30	4.06	0.000	*Blautia*
	Otu43	3.92	0.000	*Collinsella*
	Otu117	3.81	0.000	*Roseburia*
	Otu137	3.78	0.000	Uncultured Lachnospiraceae
	Otu49	3.60	0.012	Uncultured Ruminococcaceae
	Otu3	3.57	0.000	*Staphylococcus*
	Otu75	3.55	0.020	*Anaerococcus*
	Otu163	3.52	0.000	*Dorea*
	Otu527	3.47	0.000	*Coprococcus*
	Otu1026	3.45	0.000	Uncultured Lachnospiraceae
	Otu22	3.38	0.000	*Lactobacillus*
	Otu1086	3.29	0.020	Uncultured Ruminococcaceae
	Otu97	3.26	0.000	*Anaerostipes*
	Otu2858	3.25	0.000	Uncultured Lachnospiraceae
	Otu165	3.12	0.000	Uncultured Lachnospiraceae
	Otu262	3.10	0.000	*Butyricicoccus*
	Otu399	3.10	0.046	Uncultured Ruminococcaceae
	Otu315	3.07	0.000	Uncultured Lachnospiraceae
	Otu3495	3.05	0.001	*Fusicatenibacter*
	Otu252	3.03	0.011	Uncultured Ruminococcaceae

The microbiota of patients treated with PBLs (*n* = 194), OBLs (*n* = 133), and FQNs (*n* = 63) was compared at D6 to identify the class-specific impact of broad-spectrum antibiotic treatment. Distinctly abundant OTUs associated with each timepoint were identified using linear discriminant analysis effect size (LEfSe, LDA > 3.0). D6: rectal swab collected ~6 days after initiation and at the end of antibiotic treatment. PBL: penicillin + beta-lactamase inhibitor. OBL: other beta-lactamase antibiotics. FQN: fluoroquinolones. LDA: linear discriminant analysis score. OTU: operational taxonomic unit.

### Large alterations in microbial composition in AAD and CDI patients

**Fig. 5:**
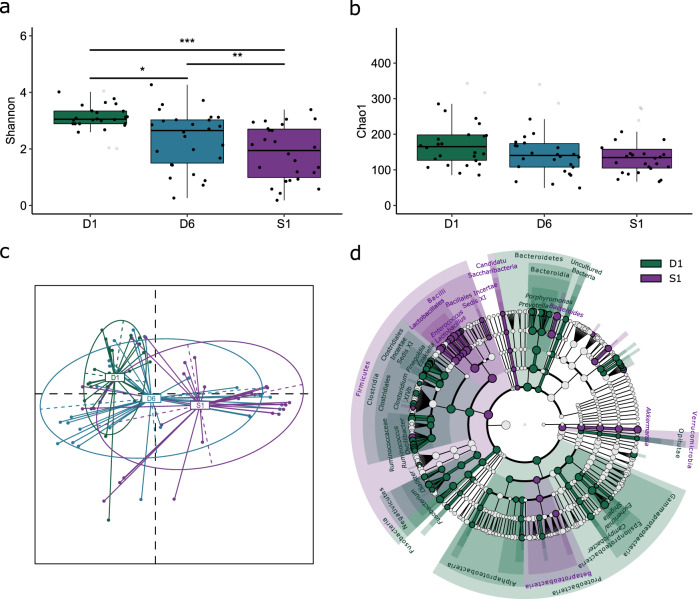
Longitudinal analysis of microbial diversity and dysbiosis in patients developing AAD. Microbial diversity and composition in patients developing AAD in the study population (*n* = 26) was assessed at D1 (green), D6 (blue), S1(purple). **a** Gradual decrease in Shannon diversity was observed between all timepoints (from D1 to D6: *p* = 0.018, from D1 to S1: *p* = 2.74*10^−5^, from D6 to S1: *p* = 0.007). **b** Similar trends are observed for the Chao1 index (Friedman rank sum: *p* = 0.054). **c** Multi-dimensional scaling (MDS) shows distinct clusters for samples collected at each timepoint. **d** Comparison of the microbiota composition at D1 and S1 conducted using LEfSe (LDA > 2.0) shows large changes in the Firmicutes and Proteobacteria phyla for AAD patients. Proteobacteria are significantly reduced at the occurrence of AAD, and a shift is observed from the Clostridia to Bacilli class at the instance of diarrhea. The cladogram shows distinct taxa of interest. For more details, see Supplementary Fig. 5. Alpha diversity indices were compared using the paired two-sided non-parametric Wilcoxon signed rank test followed by Bonferroni correction of *p*-values. Box plots indicate median (middle line), 25th, 75th percentile (box), and 5th and 95th percentile (whiskers) as well as outliers (gray single dots). AAD: patients with non-*C. difficile* antibiotic-associated diarrhea. D1: rectal swab sample collected upon study enrollment. D6: rectal swab collected ~6 days after initiation and at the end of antibiotic treatment. S1: stool sample collected at the first occurrence of diarrhea (variable time-point). LEfSe: linear discriminant analysis effect size. LDA: Linear discriminant analysis score. *p < 0.05, **p ≤ 0.01, ***p ≤ 0.001.

To better understand associations between antibiotic-induced perturbations in the intestinal microbiota and the occurrence of diarrhea, we determined alterations in alpha and beta diversity between D1, D6, and at the time of first diarrheal onset in 32 patients (S1; AAD *n* = 26, CDI *n* = 6). The median time to S1 was 6 days (Interquartile range [IQR]: 3–24) for patients with AAD. A decreasing trend in overall diversity described by the Shannon index was observed in patients with AAD between D1, D6, and S1 samples (Fig. 5a, *p* ≤ 0.018), indicating that the dysbiosis induced by antibiotic treatment worsened at the onset of diarrhea. Microbial richness described by the Chao1 index revealed a slight decrease between D1 and D6 as well as between D1 and S1 (Friedman rank sum: *p* = 0.054, Fig. 5b). Additionally, multi-dimensional scaling (MDS) revealed a shift in microbial composition between different time-points with the largest differences observed between D1 and S1 (Fig. 5c), primarily due to decreasing abundances of the Proteobacteria phylum, and the Clostridia and Bacteroidia classes. Members of the Clostridiales Incertae Sedis XI family were significantly reduced, together with *Prevotella*, *Escherichia/Shigella*, and *Finegoldia* amongst others (Fig. 5d, Supplementary Fig. 5). Further, a remarkable shift in members of the Firmicutes phylum was observed between D1 and S1 samples of AAD patients: from dominance of Clostridia to an increasing proportion of Bacilli, wherein Lactobacillales increased in relative abundance together with *Enterococcus*, *Streptococcus*, *Lactobacillus*, and *Akkermansia*. Interestingly, the increase in *Enterococcus* in the AAD-associated microbiota (S1 samples) was due to the same OTU that was identified in this study as a predictive marker of CDI development and that also increased after PBL and OBL treatments.

Due to the low number of stool samples obtained from patients with ongoing CDI, the alterations in microbial composition observed at the occurrence of CDI (S1) compared to that observed at baseline (D1) were less well-defined. However, many similarities were observed between the AAD and CDI microbiota (Supplementary Fig. 6, Supplementary Results). At the occurrence of diarrhea, both the CDI and AAD microbiota were vastly dominated by Firmicutes, whereas the abundance of Bacteroidetes and Proteobacteria was reduced. In addition, both the CDI and AAD microbiota (S1 samples) showed elevated levels of *Enterococcus* and a drastic decrease in *Prevotella* spp.

## Discussion

In this prospective study, we performed longitudinal characterization of the intestinal microbiota of 945 hospitalized patients from six European countries included in the ANTICIPATE study based on known risk factors for development of CDI. Our primary aim was to identify specific microbial markers or patterns in the intestinal microbiota predisposing to CDI and AAD that could be detected in asymptomatic patients at the time of hospital admission. We also characterized the changes associated with antibiotic treatment potentially impacting the subsequent risk of CDI and AAD development. This was achieved using 16S rRNA profiling combined with a high-resolution sequence typing approach (oligotyping), and when required, further delineation of species using shotgun metagenomic sequencing.

At the start of antibiotic treatment, intestinal microbial diversity was lowest in patients who subsequently developed CDI (median time to development: 18 days, range 1–78) and highest in patients not developing AAD or CDI. In the low-diversity microbiota of patients subsequently developing CDI, pathobionts belonging to the *Enterococcus* genus were present in significantly higher abundances compared to AAD or ND patients. Higher abundance was attributed to a single OTU classified as *Enterococcus*, and constituted primarily by *E. faecium* and *E. faecalis*, which are also the two most frequently occurring enterococci in the human intestine^32^. We further identified the same OTU in higher abundance in diarrheic stool samples of patients with CDI (Supplementary Fig. 6, Supplementary Results), as has also been repeatedly observed in previous studies analyzing stools from patients with active CDI^10,12–17^. Baseline samples of CDI patients also showed remarkably low abundances of *Blautia* (mainly *B. wexlerae*, *B. obeum*, and *B. luti*)*, Ruminococcus* (*R. torques*, *R. lactaris*, and *R. bromii*)*, Porphyromonas* (*P. bennonis* and *P. uenonis*)*, Bifidobacteria* (*B. adolescentis*, *B. catenulatum*, and *B. dentium*)*, Odoribacter*, and *Ezakiella* spp. in comparison to non-CDI patients.

In order to validate our results in independent patient cohorts, we screened the literature for studies in CDI patients with a prospective sampling design, and identified one study that had investigated the microbial composition of the intestinal microbiota in patients prior to CDI development^18^. This dataset, generated from a Canadian cohort of elderly patients who developed CDI and from non-diarrheic controls served as an independent validation to verify the generalizability and predictive potential of the microbiota-based markers identified here. Reanalysis of this decade-old Canadian dataset, which was generated using a different sequencing technology than that utilized in this study, also revealed a low-diversity microbiota with elevated levels of *Enterococcus* spp. (mainly *E. faecium* and *E. faecalis*) and depleted of *Ruminococcus*, *Ezakiella*, and *Odoribacter* spp. 5 days prior to development of CDI in this cohort.

These markers were dichotomized, developed into predictive OTU ratios or relative abundance thresholds, and after assessment in competing event models, the best predictive ratio was that of *Enterococcus* relative to *Ruminococcus* predictive of a 5-fold higher risk of CDI in our dataset. The best abundance-based OTU model utilized relative abundances of *Enterococcus* relative to *Alistipes* and was similarly found to be predictive of a 5-fold higher CDI risk. Both markers predicted an increased CDI risk in the Canadian cohort of 4.6 or 6.2 times, respectively, as described in van Werkhoven et al.^29^.

Remarkably, treatment with beta-lactams, both PBLs and OBLs, was also associated with an increased relative abundance of the enterococcal OTU. *Enterococcus* spp. are intrinsically resistant to most cephalosporins and clindamycin, have reduced sensitivity to staphylococcal-targeting penicillins and carbapenems, and clinical strains are frequently resistant to FQNs^32^. Since the 1950s, ampicillin has been the first choice of treatment of enterococcal infections^33^. Yet, since 2000, a specific clade of *E. faecium* has become dominant in healthcare settings worldwide and is characterized by, amongst others, resistance to ampicillin^33,34^. Further, emergence of vancomycin resistance in this genus, especially in the *E. faecium* species, has rendered it the ultimate hospital-adapted organism^34^. Prior studies have shown that vancomycin-resistant enterococci (VRE) colonization of the intestinal microbiota may protect against acquisition of common enteric pathogens such as *E. coli* and GI viruses, and predispose to increased *C. difficile* infections^35,36^. Therefore, it is possible that the increased colonization with VREs as a result of beta-lactam therapy heightens the risk of CDI development.

A comparison of baseline (D1) samples from patients developing CDI and AAD showed lowest diversity in patients developing CDI followed by those developing AAD, compared to ND patients as well as the presence of distinctive microbial markers with *Enterococcus* spp. and Clostridiales Incertae Sedis XI, respectively, as being the most characteristic. In contrast, stool samples from patients with active CDI and AAD exhibited many similarities, including depletion of *Blautia* spp. and *Prevotella* spp., and an increased enterococcal abundance as also observed by previous studies^10,12,14,15,19^. These data indicate a convergent evolution towards a diarrheic, dysbiotic microbiota and also suggest that *C. difficile* might not be the driver underlying the changes observed in the microbiota, but rather opportunistically invades an antibiotic-induced low-diversity microbiota abundant in *Enterococcus* spp. The latter hypothesis is supported by the lack of *C. difficile* carriage in D1 (10/14) and D6 (12/14) samples of patients later developing CDI in this study. Although the prevalence of *C. difficile* carriage was similar after antibiotic treatment (D6), relative abundances of the organism were distinctly higher compared to those at D1. Expectedly, *C. difficile* prevalence identified by 16S rRNA-based techniques in the present study was higher than that of toxigenic *C. difficile*, identified by Xpert^®^*C. difficile*/Epi panel (Cepheid, CA, USA) in the same set of patients^29^, as the former do not discriminate between toxigenic and non-toxigenic*C. difficile*. Of note, the majority of patients harboring *C. difficile* at D6 were distinct from *C. difficile*-positive patients at D1, indicating potential nosocomial acquisition of the organism upon or during antibiotic treatment.

In addition to an overall decrease in alpha diversity after treatment with PBLs, OBLs, and FQNs, we identified distinct changes in microbiota profiles at D6 linked to each of the three antibiotic classes. Notwithstanding the enterococcal bloom following beta-lactam therapy, PBL treatment further led to a reduction in Clostridiales Incertae Sedis XI members. The Clostridiales Incertae Sedis XI bacterial family has been previously associated with decreased CDI risk, as a decrease in relative abundance prior to onset of CDI has been reported^18^. Here we show that this decrease, resulting from PBL treatment, was observed in the D6 samples of patients who eventually developed CDI, AAD, or did not develop diarrhea. OBL treatment led to large reductions in members of the Lachnospiraceae family comprising producers of butyrate, a short-chain fatty acid associated with intestinal health^37^, suggesting that a reduction might render patients more susceptible to CDI. Both beta-lactam and FQN treatment led to a decrease in specific *Prevotella* spp. that showed higher abundance in the baseline ND microbiota and were remarked to be depleted at the time of occurrence of AAD. Collectively, all investigated antibiotic classes significantly altered taxa with documented links to the development or occurrence of CDI and AAD further underscoring their role as high-risk antibiotics.

One of the main challenges faced by microbiota studies is the variability between patients in the study population as no two patients have the exact same baseline microbiota profile. This makes evaluation of specific changes difficult and highlights the need for longitudinal sampling, which is one of the strengths of our study. The low CDI incidence in hospitalized patients was considered when designing this study by limiting recruitment to patients exposed to known risk-factors for CDI, such as broad-spectrum antibiotic treatment. Despite such efforts, the observed CDI incidence was lower than anticipated, and constitutes a limitation of this study. Nonetheless, as shown by the independent validation study performed on patient samples collected a decade prior and from a different continent, we successfully identified robust microbial markers predictive of the development of CDI that prevail over confounders commonly limiting the wider applicability of microbiota-based markers. Future applications include enrichment of high-risk patients in prospective clinical trials, development of predictive, microbiota-based diagnostics to tailor antibiotic therapy or stool biobanking from high-risk patients prior to antibiotic therapy, exemplifying a precision medicine approach.

## Methods

### Study design

Samples used in this study were collected in ANTICIPATE (ClinicalTrials.gov *NCT02896244*), a multi-center prospective observational study conducted at 34 European hospitals in Germany, Greece, France, Romania, Spain, and the Netherlands. Ethical approval was obtained at each participating site in accordance with local regulations (Supplementary Information) and written informed consent was obtained from all participants prior to any study-related procedures. Inclusion criteria, overall patient characteristics, diagnostic algorithms, etc., are described in detail in van Werkhoven et al.^29^. Briefly, 1007 hospitalized patients aged 50 years or above, receiving one or several antibiotics of either penicillins with a beta-lactamase inhibitor, 3rd or 4th generation cephalosporins, clindamycin, carbapenems, or FQNs were enrolled between September 2016 and October 2017 (Fig. 1). Three rectal swabs per patient were collected: two were collected at study enrollment prior to or within the first 6 h after the start of antibiotic treatment (D1) where one was used for detection of toxigenic *C. difficile* carriage using the Xpert^®^*C. difficile*/Epi panel (Cepheid, CA, USA) as described in van Werkhoven et al.^29^; the third after 6 days ± 24 h after the start of antibiotic treatment (D6), or at hospital discharge.

In this study, the second sample collected at D1 paired with the sample collected at D6 were utilized for fecal microbiota analysis as described below. In case of diarrhea, defined as loose stool scoring 5–7 on the Bristol Stool chart^38^ with ≥3 discharges within 24 h, a stool sample was collected for CDI testing during each episode during the course of the entire 90-day study period. CDI diagnosis was performed as described by the ESCMID guidelines^39^. AAD was defined as non-*C. difficile* diarrhea confirmed by a negative diagnosis.

### Population and sample characteristics

Baseline characteristics were evaluated by comparing samples grouped by age in decades, country of origin, gender, reason for hospitalization, hospitalization ward, and comorbidities to study the impact on the intestinal microbiota. To identify potential biomarkers of CDI and AAD development, samples collected at D1 were divided into three groups based on patient outcome; samples originating from patients who developed CDI (*n* = 14), confirmed non-*C. difficile* AAD (*n* = 64), and non-diarrheic patients (*n* = 669) who completed the 90-day study period, referred to as CDI, AAD, and ND patients, respectively. Patients prematurely lost to follow-up due to withdrawal of consent (*n* = 81) or death (*n* = 77), and patients with diarrheal episodes that did not undergo CDI testing (*n* = 40) were excluded for this analysis. Studies related to the specific effect of different antibiotics on the microbiota were conducted on samples from patients who provided both a D1 and a D6 sample (*n* = 737). These were divided into groups according to received antibiotics classified according to the 2019 ATC index (WHO Collaborating Centre for Drug Statistics Methodology, https://www.whocc.no/) based on their mode of action and chemistry. All antibiotics received between study enrollment (D1) and up to one day prior to collection of the second rectal swab at D6 were considered for classification. Patients who received antibiotics belonging to only one class, irrespective of number of treatments, number of different antibiotics received within the same class, or duration, were considered for analysis whereas patients receiving combination treatment or treatment with antibiotics belonging to more than one class were excluded. The three studied antibiotic classes were PBLs (namely penicillins with extended spectrum); OBLs (namely cephalosporins and carbapenems); and FQNs. In cases where stool samples were sequenced, temporal analysis of patients who developed CDI and AAD was conducted at D1, D6, and the onset of the first diarrheal episode (S1).

### Sample collection and DNA isolation

Rectal swab samples were collected by inserting swabs (FecalSwab Regular Flocked Collection Kit, Copan Diagnostics Inc., Murrieta, USA) 2 cm into the rectum in a rotating manner and stored at –80 °C within 4 h of collection. In case of diarrhea, a stool sample was collected for CDI testing during each episode by patients and transported under refrigerated conditions to the local hospital lab and subsequently stored at –80 °C. Total metagenomic DNA was extracted from thawed fecal samples using the FastDNA SPIN Kit (MP Biomedicals, Santa Ana, USA) according to the manufacturer’s instructions. Each DNA extraction batch was processed with a blank swab as a negative control. DNA quantity was assessed by using the Qubit ds DNA HS Assay Kit with a Qubit 3.0 Fluorometer (ThermoFisher Scientific, Waltham, USA). In case of insufficient DNA concentration for library preparation, samples were concentrated using a Savant DNA120 SpeedVac (Thermo Scientific, Waltham, USA) and dissolved in 30 μl double-distilled water.

### 16S rRNA gene sequencing

16S rRNA gene libraries were prepared using the Nextera XT kit (Supplementary Methods, Supplementary Table 11) and sequenced using 2 × 250 or 2 × 300 paired-end sequencing as described by the manufacturer (Illumina Inc., San Diego, USA). The average number of raw reads in the sequenced samples was 80,145 (range 1–1,343,956) with a sequencing error rate of 0.01% (Supplementary Fig. 8). In case of failed sequencing due to low sample concentration, negative PCRs, low number of reads (<20,000 raw or <15,000 processed reads, Supplementary Fig. 8), or poor read quality (Phred score < 25, Supplementary Fig. 9), sequencing was repeated. DNA from four pure *C. difficile* isolates, mock communities consisting of pooled 16S rRNA gene sequences of 20 bacterial strains (HM-783D, https://www.beiresources.org/), and extracted DNA from previously sequenced samples were included as positive sequencing controls, together with negative PCR and DNA extraction controls to ensure sequencing reproducibility (Supplementary Figs. 11–13).

### 16S rRNA gene profiling

#### Pre-processing, quality filtering, and classification

Data pre-processing was performed using the OCToPUS v1.0 pipeline^40^, which starts by de-noising each of the forward and reverse raw reads separately using the implemented k-mer frequency in SPAdes v3.5.0^41^. Contigs were created by heuristically merging de-noised paired-end reads in mothur v.1.39.1^42,43^. Contigs with base ambiguities, representing Phred scores below 25 or unresolved mismatches between forward and reverse reads, were discarded, whereas the remaining contigs were aligned to the SILVA database v.119^44^. Contigs aligning outside the V3–V4 region were discarded together with contigs containing more than eight homopolymers, and with unexpected length. Denoising of the trimmed sequence alignment was conducted with the Illumina Paired-End Denoiser (IPED) algorithm v1.0^45^ followed by de novo chimera removal with CATCh v1.0^46^. OTU clustering was performed with UPARSE (USEARCH v8.1.186 implementation) at a 97% identity level^47^, followed by taxonomic classification against the Ribosomal Database Project (RDP) dataset v. 16 using the default cutoff of 80%^48^.

#### Primary data analysis

Further stratification of OTU membership was conducted for OTUs of interest, such as the OTU classified as *Clostridium XI*, which contains *C. difficile*, using oligotyping v2.2^49^. Oligotyping can distinguish down to single-nucleotide differences in ribosomal 16S rRNA sequences between species while disregarding sequencing errors. Identified oligotypes were classified with NCBI Nucleotide BLAST using default settings and the Ribosomal 16S rRNA database as reference. Only hits with an identity >97% were considered. Rarefaction curves were calculated in mothur, and a sequencing depth of 15,000 reads was chosen as a tradeoff between coverage and number of discarded samples and was applied to all samples before further analysis (Supplementary Fig. 9). *C. difficile* carriage was determined in all sequenced samples by oligotyping. Alpha diversity indices Shannon and Chao1 were calculated in mothur together with beta diversity described by the Jaccard index chosen specifically for its enhanced sensitivity to rare OTUs compared to other weighted indices.

#### Statistical analysis and visualization

Statistical comparison of the alpha diversity was conducted using two-sided non-parametric Kruskal–Wallis or Wilcoxon signed-rank tests for non-paired comparisons, and paired Friedman rank sum or Mann–Whitney tests as indicated. In case of multiple-comparison testing, such as when comparing microbial diversity at several timepoints collected from the same patient or when comparing diversity between antibiotic groups at D6, Bonferroni correction of *p*-values was performed. Analysis of molecular variance (AMOVA), an ANOVA-like statistical method developed for metagenomic datasets, was used to compare beta diversity between samples. Further analysis and visualization was performed using the *ggplot2* v3.3.2 and Rhea v.1.6 packages in RStudio v. 3.5.0^50,51^. Microbial biomarker prediction and identification of significant OTUs (*p* < 0.05) using Linear Discriminant Analysis Effect Size (LEfSe) v1.0.0 with an LDA score > 2.0 was performed in mothur^52^. For longitudinal analysis of microbial changes as a result of antibiotic treatment, an LDA score of >3.0 was used to identify the most relevant changes. *p*-values < 0.05 were considered significant for all analyses. Only OTUs with an average relative abundance >0.1% were reported for the LEfSe analyses. The relationship between *C. difficile* carrier at D1 and D6 were visualized using Circos^53^.

#### Microbiota-based biomarker validation

To assess robustness of the identified microbiota-based biomarkers in this study, biomarker detection was performed in an independent validation dataset^18^. These data (*n* = 50) were collected from hospitalized patients aged ≥18 years in 2005–2006 in Canada and contains prospectively collected fecal samples obtained within the first 7 days of hospital admission. Patients who developed CDI (*n* = 25) within the 60-day follow-up period were matched with control patients (*n* = 25) based on age, gender, and hospitalization date. Library preparation and sequencing of the V3–V5 regions of the rRNA gene in this study was conducted using 454 Pyrosequencing.

Raw sequencing data were processed similarly to MiSeq data generated in this study. First the reads with base ambiguities or homopolymers more than eight bases were discarded, then aligned to the V3–V4 region of the SILVA database v.119^44^, discarding reads failing to fulfill the expected length. Denoising and chimera removal was performed using mothur and CATCh^46^, respectively. Lastly, reads were clustered to the same set of OTUs (excluding rare OTUs with abundance < 0.1%) with UPARSE at a 97% identity level^47^, followed by taxonomic classification against the RDP dataset v. 16. One sample containing only 32 processed reads was removed due to low coverage prior to further analysis. To identify microbiota-based markers associated with CDI, microbial composition of samples collected from ND and CDI patients were compared using LEfSe (*p* < 0.05, LDA > 2.0). Identified OTUs were further oligotyped as previously described.

### Shotgun metagenomic and sequencing

Available DNA quantity of D1 samples originating from patients who developed CDI in this study (*n* = 14) was assessed, and where sufficient amounts remained (*n* = 9), shotgun metagenomic DNA libraries were prepared using the Nextera XT kit followed by 2 × 150 bp paired-end sequencing with a NovaSeq instrument (Illumina Inc., San Diego, USA) with at least 1,000,000 raw reads per sample according to the manufacturer’s instructions.

### Analysis of shotgun metagenomic data

Raw read quality was assessed using FastQC v0.11.9 (https://www.bioinformatics.babraham.ac.uk/projects/fastqc/), followed by adapter trimming using Trim Galore v0.6.4 (https://github.com/FelixKrueger/TrimGalore). Differentially abundant OTUs defined by LEfSe when comparing patients who developed CDI follow-up with all others, and their respective oligotypes classified using NCBI blast against the 16S database, were used as a basis for taxonomical assignment of shotgun metagenomic reads. A manually curated Kraken2 database was constructed using representative genomes obtained from NCBI containing only those species listed in Supplementary Table 6 (Supplementary Table 12). Subsequent taxonomic assignment of trimmed reads was performed using Kraken 2^54^ v2.0.9 with a confidence score of 1 using the manually curated database as reference.

### Reporting summary

Further information on research design is available in the Nature Research Reporting Summary linked to this article.

## Supplementary information

Supplementary Information

Reporting Summary

## Data Availability

Data supporting the findings of this study are available within the paper and its Supplementary Information file. 16S rRNA and shotgun metagenomic sequence data generated and analyzed in this study have been deposited in the NCBI Sequence Read Archive with the accession code PRJNA685914. Human reads were identified and removed prior to shotgun metagenomics data upload. All other data generated in this study are available from the corresponding author upon reasonable requests. Raw data from Vincent et al.^18^ utilized as a validation cohort in this study was kindly provided by Prof. Amee Manges (University of British Columbia, Vancouver, Canada). The following public databases were utilized for analysis in this manuscript: SILVA v.119 (https://mothur.org/wiki/silva_reference_files/), Ribosomal Database Project (RDP, https://mothur.org/wiki/rdp_reference_files/) v.16. Source data are provided with this paper.

## Source data

Source Data
