# Supplementary material for: Microbiota-based markers predictive of development of Clostridioides difficile infection
Source: Nat Commun. 2021 Apr 14;12:2241. doi: 10.1038/s41467-021-22302-0 (PMC8047037; doi:10.1038/s41467-021-22302-0)
Supplement: Supplementary file 1 — Supplementary Information [file 41467_2021_22302_MOESM1_ESM.pdf]

Supplementary Information

Microbiota-based markers predictive of development of *Clostridioides difficile* infection

Matilda Berkell[1], Mohamed Mysara[1,2], Basil Britto Xavier[1], Cornelis H. van Werkhoven[3], Pieter Monsieurs[2,4], Christine Lammens[1], Annie Ducher[5], Maria J.G.T. Vehreschild[6,7,8], Herman Goossens[1], Jean de Gunzburg[5], Marc J.M. Bonten[3,9], Surbhi Malhotra-Kumar[1*], the ANTICIPATE study group[10]

[1] Laboratory of Medical Microbiology, Vaccine & Infectious Disease Institute, University of Antwerp, Universiteitsplein 1, 2610 Wilrijk, Belgium
[2] Microbiology Unit, Interdisciplinary Biosciences, Belgian Nuclear Research Centre, SCK–CEN, Boeretang 200, 2400 Mol, Belgium
[3] Julius Center for Health Sciences and Primary Care, University Medical Center Utrecht, Utrecht University, Heidelberglaan 100, 3584 CX Utrecht, the Netherlands
[4] Department of Biomedical Sciences, Institute of Tropical Medicine, Antwerp, Belgium [5] Da Volterra, 172 rue de Charonne, 75011 Paris, France;
[6] Department of Internal Medicine, Center for Integrated Oncology Aachen Bonn Cologne Duesseldorf, University of Cologne 50924 Cologne, Germany
[7] German Centre for Infection Research (DZIF), partner site Bonn-Cologne, Germany
[8] University Hospital Frankfurt, Department of Internal Medicine, Infectious Diseases, Goethe University Frankfurt, 60590 Frankfurt am Main, Germany
[9] Department of Medical Microbiology, University Medical Center Utrecht, Utrecht University, Heidelberglaan 100, 3584 CX Utrecht, the Netherlands
[10] The ANTICIPATE study group investigators are listed at the end of the manuscript
*Corresponding author, email: surbhi.malhotra@uantwerpen.be

These authors contributed equally: Matilda Berkell, Mohamed Mysara, Basil Britto Xavier

Table of contents

Ethics committees ... 3

Supplementary Results ... 5

- Evaluation of baseline characteristics of enrolled patients ... 5
- High-resolution 16S rRNA gene sequence analysis reveals concealed diversity in an OTU classified as *Clostridium XI* ... 6
- LEfSe analyses ... 7
- Investigation into the prevalence of one OTU classified as *Sphingomonas* ... 7
- Patients developing CDI undergo large alterations in microbiota composition ... 8
- Quality controls ... 9
- Investigation into the effect of sequencing chemistry ... 10

Supplementary Methods ... 11

- PCR amplification of the 16S rRNA V3-V4 regions ... 11

Supplementary Tables ... 12

Supplementary Figures ... 30

Supplementary References ... 38

Ethics committees

Netherlands: local medical ethics committee approval

- UMC Utrecht

Germany: local medical ethics committee approvals

- UKK Uniklinik Köln
- Universitätsklinikum Heidelberg (KLIPPS)
- Jena University Hospital
- UK-SH (UZL) Universitätsklinikum Schleswig-Holstein, Campus Lübeck
- Klinikum der Universität München
- Universitätsklinikum Leibzig
- University of Aachen
- Universitatsklinikum Essen

Greece: local medical ethics committee approvals

- University Hospital of Heraklion
- Laiko General Hospital
- Attikon University General Hospital
- Evangelismos General Hospital of Athens
- Ippokrateio General Hospital of Athens

Spain: Central approvals:

- Comité Coordinador de Ética de la Investigación Biomédica de Andalucía
- Dirección General de Inspección y Ordenación CONSEJERÍA DE SANIDAD Comunidad de Madrid
- Local medical ethics committee approvals
- Hospital Universitari de Bellvitge
- Hospital Universitario 12 de Octubre
- Hospital Universitario Gregorio Marañon
- Hospital Universitario Ramon y Cajal
- Hospital Universitario Virgen Macarena
- Hospital Universitari Vall d'Hebrón
- Servicio Andaluz de Salud- Reina Sofia University Hospital

Romania: Central approval:

- Ministry of Health, National Agency for Medicines and Medical Devices
- Local medical ethics committee approvals
- Infectious and Tropical Diseases Hospital "Dr. Victor Babes"
- Clinical Hospital Of Infectious Diseases Of Iasi
- The National Institute of Infectious Diseases Matei Bals
- Cluj Napoca Infectious disease Clinical Hospital
- Oncology Institute Ion Chiricuta

France: Central approvals:

- ANSM (Agance nationale de sécurité du médicament et des produits de santé)
- Comite de protection des personnes du Sud-Ouest et outre-mer IV, Limoges

Supplementary Results

Evaluation of baseline characteristics of enrolled patients

Assessment of impact of baseline characteristics on microbial composition in hospitalized patients are summarized in this section. Many baseline characteristics such as hospitalization ward, hospitalization reason, comorbidities etc. were explored. However, it was difficult to divide patients into groups based on e.g. comorbidities as most have more than one and were thus not explored further. Similarly, other characteristics did not provide large enough groups to enable comparison and were therefore not explored further.

The largest differences were observed between patients based on country of origin and age. Overall, patients from different countries showed distinct differences in alpha diversity (Shannon: $p < 0.001$, Chao1: $p < 0.001$), where the microbial diversity in Romanian patients were particularly distinct (Supplementary Table 1). Further exploration of structural changes confirmed that Romanian patients have a unique microbial composition compared to that of countries, and a microbiota enriched in Ruminococcaceae, *Faecalibacterium*, and *Roseburia* compared to all others (Supplementary Figure 1a). When comparing patients based on gender, no distinct differences were observed in alpha diversity whereas slight compositional differences were observed (Supplementary Table 1). Male study participants displayed higher levels of Prevotellaceae, Bacteroidetes, Clostridiales Incertae Sedix XI, and *Finegoldia* whereas female participants had higher levels of Proteobacteria, Sphingomonadales, Lachnospiraceae, and Faecalibacterium (Supplementary Figure 1b).

High-resolution 16S rRNA gene sequence analysis reveals concealed diversity in an OTU classified as *Clostridium XI*

Clustering of short 16S rRNA gene sequencing reads at 97% does not guarantee single-species resolution within an OTU[1]. At this cutoff, 58.8% (n = 556) of the patients at D1 harbored reads clustered into one OTU classified as *Clostridium XI* containing all pure *C. difficile* reads verified by sequencing of positive controls. This far exceeds *C. difficile* carriage rates reported both in hospitals and the community (7–18% and 2–4%, respectively)[2]. Therefore, for high-resolution analysis it was necessary to perform oligotyping[3], which revealed that this OTU consisted of two distinct sequence types distinguished by 6 nucleotides corresponding to two organisms, *C. difficile* and *Clostridium bartlettii,* where the latter is also an anaerobic occupant of the human intestine[4]. Both organisms were identified with 100% sequence length and identity with no other closely-related hits. *C. bartlettii* is a sporulating anaerobic occupant of the human intestine with limited genome similarity to *C. difficile* but sharing some common phenotypic functions and, importantly, a 16S rRNA gene similarity of > 97% in the V3–V4 region[4–6]. Thus, when investigating single species in short-read 16S data, high-resolution analysis tools such as oligotyping[3], DADA2[7], or Resphera[8] are recommended to avoid overestimating carrier rates due to insufficient resolution in addition to previously known limitations of species delineation using short-read sequencing, such as insufficient sequencing depth and sampling techniques[8]. Overall, 82.6% of the reads in this OTU were classified as *C. bartlettii* rendering *C. bartlettii* 4.7 times more frequent than *C. difficile* in this patient cohort. Furthermore, it was found that the *C. bartlettii* / *C. difficile* read ratio within this OTU varied widely between patients (14.78 on average, SD = 35.06).

LEfSe analyses

Linear Discriminant Analysis Effect Size (LEfSe) was performed on D1 samples in order to identify possible microbiota-based markers that could be used for prediction of CDI and AAD. Three comparisons were made of the samples (Supplementary Table 2); CDI vs non-CDI patients to enable prediction of CDI (Supplementary Table 3), diarrheic vs. ND patients to enable prediction of development of antibiotic-associated diarrhea (Supplementary Table 4), and a three-way comparison of AAD, CDI, and ND patients in order to distinguish the three patient groups from each other (Table 2). The ability for prediction of the output was assessed by regression modelling with competing events as described in van Werkhoven *et. al.*[9].

Investigation into the prevalence of one OTU classified as *Sphingomonas*

In several of the investigated antibiotic groups, we observed an increase in relative abundance of *Sphingomonas* at D6. *Sphingomonas* spp. are unlikely occupants of the human intestine as this genus constitutes strictly aerobic bacteria commonly occurring in the environment. Previous reports indicate that the occurrence of this genus can be observed after 16S rRNA gene sequencing in samples with low biomass input or arise due to contaminations in PCR or DNA extraction reagent kits[10–14]. In total 42 samples were identified with a high relative abundance of this OTU in the whole sample collection. To exclude this as an introduced contamination by sample handling, samples with a high relative abundance were evaluated based on sampling site, DNA extraction date, DNA concentration, DNA extraction kit lot number, and sequencing date and batch. It was found that these samples could be linked to a few sequencing batches in which the negative controls displayed this same OTU. After an extensive evaluation of potential contaminating factors within the identified batches, no conclusive evidence was found as not all samples in any one batch showed presence of

Sphingomonas and no clear links to any day, date or DNA extraction or sequencing kit, or to any personnel handling of the samples could be identified.

<u>Patients developing CDI undergo large alterations in microbiota composition</u>

Differences in alpha diversity and microbial composition were less pronounced between sampling points for patients developing CDI compared to those who developed AAD (Supplementary Figure 6a-b), possibly due to the small number of sequenced stool samples (n = 6). Additionally, three patients developed CDI between the D1 and D6 sampling, possibly indicating an already disrupted microbiota at D1 due to CDI development. No distinct changes in alpha diversity were observed between the sampling points (Supplementary Figure 6a-b), and no distinct separation was observed between identified MDS clusters (Supplementary Figure 6c). The only taxa found to be distinctly elevated in CDI patients at the occurrence of diarrhea include cultured and uncultured members of the Lactobacillales order (Supplementary Figures 6d and 7).

Despite the lack of significant results for the CDI group, many similarities were observed between the AAD and CDI cases. At the occurrence of diarrhea, both microbiotas were vastly dominated by Firmicutes whereas the abundance of Bacteroidetes and Proteobacteria was decreased. The CDI microbiota showed higher levels of Firmicutes and lower levels of Proteobacteria compared to the AAD microbiota. The CDI microbiota additionally showed higher abundance of the genera *Clostridium XI*, *Enterococcus*, and *Lactococcus*, as well as uncultured Lachnospiraceae, and largely lower abundance of *Faecalibacterium*, *Bacteroides*, and *Prevotella* spp. AAD patients similarly suffered from high levels of *Enterococcus*, and with large reductions in *Prevotella*. AAD patients, had higher baseline levels of *Prevotella* and *Escherichia*/*Shigella* compared to CDI, which were both reduced at the time of occurrence.

CDI patients largely lacked *Escherichia/Shigella* altogether at baseline, but the abundance at the time of occurrence were similar to that of AAD. Other highly abundant genera at the instance of AAD included *Klebsiella, Lactobacillus*, and *Streptococcus*, which were all largely absent in the CDI microbiota.

Quality controls

All analyzed samples were sequenced across 14 batches which generated 80,145 raw reads on average (1-1,343,946, Supplementary Figure 8a). Pre-processing of the raw reads resulted in the removal of 21% and 35% on average for quality filtering (primer mismatches, ambiguity, post-assembly, etc.) and chimera removal, respectively (Supplementary Figure 8b). Read quality was evaluated using Phred scores (Supplementary Figure 10), which reported a high level of similarity between batches. After processing of the reads, rarefaction curves were calculated to evaluate sequencing depth (Supplementary Figure 9). A subsampling depth of 15,000 reads was selected to ensure OTU stability while minimizing the number of discarded samples for analysis. The coverage was calculated using

$$C = 1 - \frac{n_1}{N}$$

n_1 = number of OTUs, N = total number of individuals in the sample).

The estimated coverage of 99.8% of the chosen subsampling depth was considered sufficient for the analysis in this study, and subsequently applied to all samples for further analysis to avoid sample size dependencies in the diversity assessment. In addition to raw read quality controls, several control samples were included to ensure study reproducibility. Four *C. difficile* isolates were processed as positive controls which showed that > 99.0% of the reads were clustered into the same OTU classified as *Clostridium XI*. Inclusion of mock communities (HM-783D, https://www.beiresources.org/) in different sequencing batches

showed an error rate of 0.01% and a Pearson correlation of > 98.0% (Supplementary Figure 11). Negative DNA extraction controls in the form of blank transport medium was processed as negative sample controls in the sequencing reactions. These samples consisted mainly of reads classified as *Sphingomonas*, *Phenylobacterium,* or unclassified bacteria (Supplementary Figure 12). Successfully sequenced samples were re-sequenced to serve as controls in several batches (Supplementary Figure 13) all showing a Pearson correlation > 95.0% with the original sample.

Investigation into the effect of sequencing chemistry

In this study, two sequencing chemistries were utilized for the 16S rRNA gene profiling. Therefore, an investigation into potential systematic differences at the OTU level due to the V2 and V3 sequencing chemistries was conducted. First, all samples sequenced with each of the two chemistries were compared using LEfSe, which revealed that no OTU was detected to be differentially abundant between the V2 and V3 chemistries. Furthermore, some patient samples were sequenced in multiple batches in the form of positive controls to assess reproducibility and to ensure that there were no batch dependencies. Such sample controls that were sequenced with both V2 and V3 chemistry were compared, and showed a Pearson correlation of ≥ 84.7% (Supplementary Figure 14). Further, beta-diversity was compared between the sample controls using Principle Coordinate Analysis (Supplementary Figure 15). Expectedly, these did not show any tangible differences between the repeats, indicating that there is no major differences in community composition between the two sequencing technologies.

Supplementary Methods

<u>PCR amplification of the 16S rRNA V3-V4 regions</u>

PCR was performed with 400 nM each of 341F and 802R primers with Illumina overhang adapters (Supplementary Table 11), in a total volume of 25 µl together with 2 µl extracted DNA, 0.03 µl 15 U/µl Super Taq Polymerase (SphaeroQ, Gorinchem, the Netherlands), and 2.5 µl 10x Super Taq buffer with 15 mM MgCl2 (SphaeroQ, Gorinchem, the Netherlands) amplified with the temperature profile: 95°C for 5 min, followed by 25 cycles of 95°C for 30 s, 60°C for 30 s, 72°C for 1 min, and final extension at 72°C for 10 min. Successful amplification was confirmed with electrophoresis in 1.5 % agarose gel at 150 V for 50 min. In case of a negative PCR, samples were excluded from library preparation and the PCR repeated. Libraries were prepared with the Nextera XT kit (Illumina Inc., San Diego, USA) of and sequenced with 2 x 250 or 2 x 300 paired-end sequencing using V2 or V3 chemistry on a MiSeq instrument (Illumina Inc., San Diego, USA).

Supplementary Tables

Supplementary Table 1: **Assessment of the impact of baseline characteristics on microbial diversity**

		Alpha diversity		Beta diversity
Hypothesis	Statistical test	Shannon p-value	Chao1 p-value	AMOVA p-value
Gender				
F-M	Wilcoxon rank sum	NS	NS	<0.001
Country of origin				
FR-GE-GR-NL-RO-SP	Kruskal-Wallis	1.12E-05	9.99E-07	<0.001
FR-GE	Wilcoxon rank sum	NS	2.21E-04	<0.001
FR-GR	Wilcoxon rank sum	NS	NS	<0.001
FR-NL	Wilcoxon rank sum	NS	NS	0.023
FR-RO	Wilcoxon rank sum	0.034	NS	<0.001
FR-SP	Wilcoxon rank sum	NS	NS	<0.001
GE-GR	Wilcoxon rank sum	NS	NS	<0.001
GE-NL	Wilcoxon rank sum	NS	NS	NS
GE-RO	Wilcoxon rank sum	8.18E-05	1.03E-05	<0.001
GE-SP	Wilcoxon rank sum	NS	NS	<0.001
GR-NL	Wilcoxon rank sum	NS	NS	0.014
GR-RO	Wilcoxon rank sum	NS	NS	<0.001
GR-SP	Wilcoxon rank sum	NS	NS	0.001
NL-RO	Wilcoxon rank sum	NS	NS	<0.001
NL-SP	Wilcoxon rank sum	NS	NS	0.016
RO-SP	Wilcoxon rank sum	1.85E-05	0.005	<0.001
Age by decade				
50-60-70-80-90	Kruskal-Wallis	0.034	NS	<0.001
50-60	Wilcoxon rank sum	0.032	-	0.001
50-70	Wilcoxon rank sum	NS	-	<0.001
50-80	Wilcoxon rank sum	NS	-	<0.001
50-90	Wilcoxon rank sum	NS	-	0.005
60-70	Wilcoxon rank sum	NS	-	NS
60-80	Wilcoxon rank sum	NS	-	<0.001
60-90	Wilcoxon rank sum	NS	-	NS
70-80	Wilcoxon rank sum	NS	-	0.014
70-90	Wilcoxon rank sum	NS	-	NS
80-90	Wilcoxon rank sum	NS	-	NS

Differences in alpha and beta diversity between patients in relation to baseline characteristics were assessed at D1 for enrolled patients in the study (n = 945). Specifically, differences in alpha diversity by gender, country of origin, and age by decades were assessed using non-parametric Kruskal-Wallis or two-sided Wilcoxon rank sum tests followed by Bonferroni correction of p-values as appropriate. Differences in microbial composition were determined using AMOVA based on the Jaccard beta-diversity index. Only p-values < 0.05 are reported. NS: Non-significant. -: Comparison not performed due to non-significant Kruskal-Wallis

comparison. M: Male. F: Female. FR: France. GE: Germany. GR: Greece. NL: the Netherlands. RO: Romania. SP: Spain. 50: patients aged 50-59. 60: patients aged 60-69. 70: patients aged 70-79. 80: patients aged 80-89. 90: patients aged 90-99. D1: rectal swab sample collected upon study enrollment. AMOVA: Analysis of molecular variance.

Supplementary Table 2: **Patient groups used for microbiota-based biomarker identification at D1**

CDI vs. non-CDI	CDI	14
	Non-CDI	733
	Excluded*	198
Diarrhea vs. ND	Diarrhea	129
	ND	669
	Excluded*	147
AAD vs. CDI vs. ND	AAD	64
	CDI	14
	ND	669

*: Patients excluded due to loss of follow-up or lack of CDI testing where required. AAD: patients with non-*C. difficile* antibiotic-associated diarrhea. CDI: patients with confirmed *C. difficile* infection. ND: non-diarrheic patients. Non-CDI: patients with non-*C. difficile* diarrhea or non-diarrheic patients. Diarrhea: patients with diarrhea regardless of testing of CDI status. D1: rectal swab sample collected upon study enrollment.

Supplementary Table 3: **Identification of microbiota-based biomarkers in patients developing CDI compared to non-CDI patients at D1**

Class	OTU	LDA	p-value	Taxonomy
CDI	Otu1	4.27	0.0001	*Enterococcus*
	Otu9	4.32	0.0200	*Finegoldia*
Non-CDI	Otu30	3.83	0.0076	*Blautia*
	Otu31	3.53	0.0059	*Ruminococcus2*
	Otu56	3.77	0.0253	*Porphyromonas*
	Otu280	3.53	0.0411	*Bifidobacterium*
	Otu64	3.59	0.0320	*Ezakiella*
	Otu69	3.63	0.0164	*Porphyromonas*
	Otu68	3.35	0.0379	*Alistipes*
	Otu648	3.29	0.0156	*Blautia*
	Otu106	3.18	0.0117	Uncultured Clostridiales
	Otu2540	3.40	0.0263	*Ruminococcus*
	Otu137	3.14	0.0487	Uncultured Lachnospiraceae
	Otu974	3.02	*0.0032*	*Roseburia*
	Otu262	2.84	*0.0020*	*Butyricoccus*
	Otu2858	2.85	0.0272	Uncultured Lachnospiraceae
	Otu294	2.79	0.0403	Uncultured Lachnospiraceae
	Otu300	2.79	0.0496	*Odoribacter*
	Otu399	2.86	0.0214	Uncultured Ruminococcaceae

The microbiota of patients developing CDI (n = 14) and of non-CDI patients (n = 733) was compared to identify predictive biomarkers at D1. Distinctly abundant OTUs associated with each group was identified using linear discriminant analysis effect size (LEfSe, LDA > 2.0).

CDI: patients with confirmed *C. difficile* infection. Non-CDI: patients with non-*C. difficile* diarrhea or non-diarrheic patients. D1: rectal swab sample collected upon study enrollment. LDA: Linear discriminant analysis score. OTU: Operational taxonomic unit.

Supplementary Table 4: **Identification of microbiota-based biomarkers in diarrheic patients compared to ND patients at D1**

Class	OTU	LDA	p-value	Taxonomy
Diarrhea	Otu1	3.58	0.0034	*Enterococcus*
	Otu18	3.42	0.0324	*Prevotella*
	Otu21	3.23	0.0046	Uncultured Lachnospiraceae
	Otu1392	3.31	0.0115	Clostridium XIVa
	Otu122	3.38	0.0301	*Veillonella*
	Otu162	2.47	0.0092	*Clostridium XVIII*
ND	Otu25	3.66	0.0133	*Campylobacter*
	Otu37	3.58	0.0486	Uncultured Clostridiales
	Otu56	3.48	0.0037	*Porphyromonas*
	Otu252	2.77	0.0210	*Ruminococcaceae*
	Otu60	2.86	0.0443	*Mobiluncus*
	Otu64	3.11	0.0159	*Ezakiella*
	Otu69	3.21	0.0049	*Porphyromonas*
	Otu92	3.24	0.0042	*Dialister*
	Otu648	3.02	0.0071	*Blautia*
	Otu106	2.76	0.0071	Uncultured Clostridiales
	Otu2540	3.02	0.0164	*Ruminococcus*
	Otu127	3.13	0.0023	*Oscillibacter*
	Otu708	2.51	0.0437	*Blautia*
	Otu527	2.76	0.0089	*Coprococcus*
	Otu974	2.74	0.0066	*Roseburia*
	Otu399	2.74	0.0015	Uncultured Ruminococcaceae

The microbiota of patients developing diarrhea (n = 129) and of ND patients (n = 669) was compared to identify predictive biomarkers at D1. Distinct OTUs associated with each condition were identified using linear discriminant analysis effect size (LEfSe). An LDA > 2.0 and $p < 0.05$ was considered significant. ND: non-diarrheic patients. Diarrhea: patients with diarrhea regardless of testing of CDI status. D1: rectal swab sample collected upon study enrollment. LDA: Linear discriminant analysis score. OTU: Operational taxonomic unit.

Supplementary Table 5: **Identification of microbiota-based biomarkers for CDI patients compared to AAD patients at D1**

Class	OTU	LDA	p-value	Taxonomy
CDI	Otu1	4.21	0.007057	*Enterococcus*
AAD	Otu9	4.29	0.021522	*Finegoldia*
	Otu30	3.79	0.030027	*Blautia*
	Otu31	3.83	0.019412	*Ruminococcus2*
	Otu280	3.59	0.038071	*Bifidobacterium*
	Otu262	2.76	0.009996	*Butyricoccus*

The microbiota of patients developing CDI (n = 14) and AAD (n = 64) was compared to identify predictive biomarkers at D1. Distinctly abundant OTUs associated with each condition were identified using linear discriminant analysis effect size (LEfSe). An LDA > 2.0 and $p < 0.05$ was considered significant. AAD: patients with non-*C. difficile* antibiotic-associated diarrhea. CDI: patients with confirmed *C. difficile* infection. D1: rectal swab

sample collected upon study enrollment. LDA: Linear discriminant analysis score. OTU: Operational taxonomic unit.

Supplementary Table 6: **BLAST results of sequences identified through oligotyping**

OTU	LEfSe	Oligotype	Genus	Species	NCBI blast % identity	Distribution between oligotypes (%)
OTU1	*Enterococcus*	1	*Enterococcus*	*hirae*	100.00	68.01
			Enterococcus	*villorum*		
			Enterococcus	*ratti*		
			Enterococcus	*faecium*		
			Enterococcus	*durans*		
		2	*Enterococcus*	*faecalis*	100.00	31.89
OTU9	*Finegoldia*	1	*Finegoldia*	*magna*	100.00	98.54
OTU30	*Blautia*	1	*Blautia*	*wexlerae*	100.00	57.84
		2	*Blautia*	*obeum*	99.75	21.51
		3	*Blautia*	*faecis*	100.00	20.42
OTU31	*Ruminococcus2*	1	*Ruminococcus*	*torques*	99.50	61.32
		2	*Ruminococcus*	*faecis*	100.00	25.39
		3	*Clostridium*	*glycyrrhizinilyticum*	99.26	4.48
		4	*Ruminococcus*	*lactaris*	99.75	8.34
OTU56	*Porphyromonas*	1	*Porphyromonas*	*bennonis*	99.53	40.54
OTU64	*Ezakiella*	1	*Bacteroides*	*coagulans*	100.00	98.28
OTU68	*Alistipes*	1	*Alistipes*	*onderdonkii*	100.00	49.26
		2	*Alistipes*	*finegoldii*	100.00	31.58
		3	*Alistipes*	*timonensis*	97.87	12.60
			Alistipes	*onderdonkii*		
OTU69	*Porphyromonas*	1	*Porphyromonas*	*asaccharolytica*	100.00	34.26
		2	*Porphyromonas*	*uenonis*	99.53	28.37
OTU280	*Bifidobacterium*	1	*Bifidobacterium*	*adolescentis*	100.00	40.91
			Bifidobacterium	*faecale*		
			Bifidobacterium	*stercoris*		
		2	*Bifidobacterium*	*pseudocatenulatum*	100.00	28.28
			Bifidobacterium	*kashiwanohense*		
			Bifidobacterium	*catenulatum*		
		3	*Bifidobacterium*	*dentium*	99.76	30.22
OTU648	*Blautia*	1	*Blautia*	*luti*	99.75	92.07
OTU106	Uncultured Clostridiales	1*	*Casaltella*	*massiliensis*	98.76–99.75	100.00
OTU137	Uncultured Lachnospiraceae	1	*Eubacterium*	*hallii*	99.00	61.00
		2	*Eubacterium*	*hallii*	99.00	35.24
OTU262	*Butyricoccus*	1	*Agathobaculum*	*butyriciproducens*	99.75	51.64
		2	*Intestinibacillus*	*massiliensis*	98.01	17.78
		3	*Agathobaculum*	*desmolans*	98.01	6.87

			Intestinibacillus	*massiliensis*		
		4	*Butyricoccus*	*faecihominis*	99.75	1.94
			Agathobaculum	*butyriciproducens*		
OTU294	Uncultured Lachnospiraceae	1	*Eubacterium*	*eligens*	94.78	96.29
OTU300	*Odoribacter*	1	*Odoribacter*	*splanchnicus*	99.52	54.64
		2	*Odoribacter*	*splanchnicus*	100.00	42.51
OTU399	Uncultured Ruminococcaceae	1*	No valid hits			100.00
OTU794	*Roseburia*	1	*Roseburia*	*inulinivorans*	100.00	95.49
OTU2540	*Ruminococcus*	1*	*Ruminococcus*	*bromii*	99.01–99.50	100.00
OTU2858	Uncultured Lachnospiraceae	1	*Dorea*	*formicigenerans*	99.00	94.78
		2	*Lactonifactor*	*longoviformis*	99.50	4.61

Sequences identified via oligotyping clustered within differentially abundant OTUs as identified by LEfSe with an average relative abundance > 0.1% were classified using NCBI blastn and the 16S rRNA database as reference where hits with an identity > 97% were reported. *: Several oligotypes detected within the OTU with the same classification. LEfSe: Linear discriminant analysis effect size. OTU: Operational taxonomic unit.

Supplementary Table 7: **Relative abundances within oligotypes defined by shotgun metagenomics**

			Relative abundance within oligotype by shotgun metagenomics									Relative abundance of oligotype by shotgun metagenomics									Relative abundance of oligotype by 16S gene profiling								
Species	OTU	Oligotype	P0022D1	P0085D1	P0254D1	P0380D1	P0603D1	P0643D1	P0715D1	P0745D1	P0754D1	P0022D1	P0085D1	P0254D1	P0380D1	P0603D1	P0643D1	P0715D1	P0745D1	P0754D1	P0022D1	P0085D1	P0254D1	P0380D1	P0603D1	P0643D1	P0715D1	P0745D1	P0754D1
Enterococcus durans			5.56	13.77	0.14	0.15	3.15	0.00	51.58	1.02	14.77																		
Enterococcus faecium			1.39	40.58	98.97	97.47	94.16	3.39	13.12	93.62	68.18																		
Enterococcus hirae	Otu1	1	0.00	8.51	0.02	0.00	0.09	0.00	0.00	1.88	0.00	0.89	9.58	99.79	86.04	99.43	28.57	60.88	87.62	90.72	2.72	51.39	100	86.49	99.42	64.85	0.00	98.03	100
Enterococcus ratti			0.00	0.00	0.00	0.00	0.00	0.00	0.00	0.00	0.00																		
Enterococcus villorum			93.06	37.14	0.87	2.38	2.60	96.61	35.29	3.48	17.05																		
Enterococcus faecalis		2	100.00	100	100	100	100	100	100	100	100	99.11	90.42	0.21	13.96	0.57	71.43	39.12	12.38	9.28	97.28	48.61	0.00	13.51	0.58	35.15	100	1.97	0.00
Finegoldia magna	Otu9	1	100.00	100	-	-	100	100	100	100	100	100	100	-	-	100	100	100	100	100	99.32	100	100	-	100	100	-	-	-
Blautia wexlerae		1	100.00	100	100	100	100	100	100	100	100	65.17	100	33.88	64.71	14.04	86.89	96.43	67.92	79.12	99.32	100	100	-	100	100	-	-	-
Blautia obeum	Otu30	2	100.00	-	100	100	100	-	100	100	100	29.53	0.00	61.16	17.65	70.18	0.00	0.72	9.43	1.05	25.87	0.00	-	-	50.00	0.00	0.00	12.50	8.28
Blautia faecis		3	100.00	-	100	100	100	100	100	100	100	5.30	0.00	4.96	17.65	15.79	13.11	2.85	22.64	19.83	4.89	0.00	-	-	20.83	0.00	0.51	0.00	16.91
[Ruminococcus] torques		1	100.00	100	-	100	100	-	100	100	100	1.56	100	0.00	99.68	90.00	0.00	99.64	22.22	40.12	69.10	100	-	-	29.17	100	99.49	87.50	74.73
[Ruminococcus] faecis	Otu31	2	100.00	-	100	100	100	-	100	100	100	3.15	0.00	75.00	0.32	6.67	0.00	0.13	77.78	58.88	25.87	0.00	-	-	50.00	0.00	0.00	12.50	8.28
[Ruminococcus] lactaris		4	100.00	-	100	-	100	100	100	-	100	95.29	0.00	25.00	0.00	3.33	100	0.22	0.00	0.99	4.89	0.00	-	-	20.83	0.00	0.51	0.00	16.91
Porphyromonas bennonis	Otu56	1	100.00	-	100	-	100	-	100	100	100	100	-	100	-	100	-	100	100	100	-	-	-	0.00	0.00	-	0.00	0.00	100
Alistipes onderdonkii		1	100.00	100	100	100	100	100	100	-	100	86.27	98.00	68.72	31.31	71.00	16.46	3.41	0.00	26.46	85.44	-	100	100	94.35	-	-	-	16.32
Alistipes finegoldii	Otu68	2	100.00	100	100	100	100	100	100	100	100	13.33	2.00	31.28	67.86	28.76	82.46	88.07	100	73.33	2.72	-	0.00	0.00	4.52	-	-	-	10.88
Alistipes timonensis		3	100.00	-	-	100	100	100	100	-	100	0.40	0.00	0.00	0.83	0.24	1.08	8.52	0.00	0.21	11.83	-	0.00	0.00	1.13	-	-	-	72.80
Porphyromonas asaccharolytica	Otu69	1	100.00	-	100	-	100	-	100	100	100	0.76	-	0.04	0.00	9.17	0.00	14.94	58.54	2.64	-	-	-	-	0.00	0.00	0.00	-	0.00
Porphyromonas uenonis		2	100.00	-	100	100	100	100	100	100	100	99.24	-	99.96	100	90.83	100	85.06	41.46	97.36	-	-	-	-	99.59	100	0.00	-	100
Anaerobutyricum hallii	Otu137	1	100.00	-	-	-	100	100	100	100	100	100	-	-	-	100	100	100	100	100	100	-	-	-	0.00	-	-	0.00	95.77
Agathobaculum butyriciproducens	Otu262	1	100.00	-	100	100	100	100	100	100	100	25.05	0.00	0.09	0.82	0.10	0.65	2.07	7.41	4.62	91.98	-	-	100	-	-	86.05	100	-
Intestinibacillus massiliensis		2	100.00	100	100	100	100	100	100	100	100	5.12	100	0.10	1.84	0.25	0.92	8.78	14.81	12.51	8.02	-	-	0.00	-	-	13.95	0.00	-

Agathobaculum desmolans		3	100.00	-	100	100	100	100	100	100	100	69.83	0.00	99.81	97.34	99.66	98.43	89.15	77.78	82.87	0.00	-	-	0.00	-	-	0.00	0.00	-
Bifidobacterium adolescentis		1	100.00	100	-	-	-	100	100	100	100	43.25	100	-	-	0.00	100	97.53	96.71	3.70	100	-	-	-	0.00	-	100	8.11	-
Bifidobacterium catenulatum	Otu280	2	90.78	-	-	-	20.00	-	100	-	97.22	55.95	0.00	-	-	83.33	0.00	0.99	0.00	95.24	0.00	-	-	-	100	-	0.00	10.81	-
Bifidobacterium pseudocatenulatum			9.22	-	-	-	80.00	-	0.00	-	2.78																		
Bifidobacterium dentium		3	100.00	-	-	-	100	-	100	100	100	0.79	0.00	-	-	16.67	0.00	1.48	3.29	1.06	0.00	-	-	-	0.00	-	0.00	81.08	-
Lachnospira eligens	Otu294	1	100.00	-	100	-	100	-	100	100	100	100	-	100	-	100	-	100	100	100	100	-	-	-	-	100	-	-	100
Odoribacter splanchnicus	Otu300	1	100.00	-	100	100	100	100	100	-	100	100	-	100	100	100	100	100	-	100	0.00	-	-	-	100	100	0.00	-	8.49
Blautia luti	Otu648	1	100.00	-	100	100	100	100	100	100	100	100	-	100	100	100	100	100	100	100	99.55	-	-	-	100	-	-	100	100
Roseburia inulinivorans	Otu794	1	100.00	100	100	100	100	100	100	100	100	100	100	100	100	100	100	100	100	100	-	-	-	-	-	-	-	-	-
Ruminococcus bromii	Otu2540	1	100.00	100	-	-	-	-	100	-	100	100	100	-	-	-	-	100.00	-	100	-	-	-	-	-	-	-	-	-
Dorea formicigenerans	Otu2858	1	100.00	100	100	100	100	100	100	100	100	80.06	100	13.49	1.08	47.15	8.96	14.87	66.67	89.71	100	100	-	-	-	-	-	100	100
Lactonifactor longoviformis		2	100.00	-	100	100	100	100	100	100	100	19.94	0.00	86.51	98.92	52.85	91.04	85.13	33.33	10.29	0.00	0.00	-	-	-	-	-	0.00	0.00

Overview of abundances of individual species within oligotypes defined by shotgun metagenomics, the total relative abundances of oligotypes identified by shotgun metagenomics, and the relative abundances of oligotypes defined by 16S rRNA gene profiling in D1 samples collected from patients developing CDI (n = 9). CDI: patients with confirmed *C. difficile* infection. D1: rectal swab sample collected upon study enrollment. OTU: Operational taxonomic unit.

Supplementary Table 8: **Oligotyping of OTUs of interest in the validation dataset**

OTU	OTU classification	Oligotype	Genus	Species	Distribution between oligotypes (%)
Otu1	*Enterococcus*	1	*Enterococcus*	*faecalis*	73.81
		2	*Enterococcus*	*hirae*<br>*villorum*<br>*ratti*<br>*faecium*<br>*durans*	26.19
Otu6	*Faecalibacterium*	1	*Faecalibacterium*	*pausnitzii*	100.00
Otu9	*Finegoldia*	1	*Finegoldia*	*magna*	100.14
Otu11	Uncultured Ruminococcaceae	1	*Ruthenibacterium*	*lactatiformans*	100.00
Otu12	*Clostridiux XI*	1	*Clostridioides*	*difficile*	68.29
		2	*Intestinibacter*	*bartlettii*	31.71
Otu14	*Prevotella*	1	*Prevotella*	*bivia*	100.00
Otu16	*Bacteroides*	1	*Bacteroides*	*thetaiotaomicron*	77.04
		2	*Bacteroides*	*finegoldii*	20.74
		3	*Bacteroides*	*xylanisolvens*	2.22
Otu26	*Akkermansia*	1	*Akkermansia*	*municiphila*	100.00
Otu28	*Peptostreptococcus*	1	*Peptostreptococcus*	*anaerobius*	86.32
		2	*Peptostreptococcus*	*stomatis*	13.68
Otu31	*Ruminococcus2*	1	*Ruminococcus*	*torques*	100.00
Otu37	Uncultured Clostridiales	1	*Fenollaria*	*massilensis*	100.00
Otu63	*Bacteroides*	1	*Bacteroides*	*uniformis*	100.00
Otu64	*Ezakiella*	1	*Bacteroides*	*coagulans*	100.00
Otu75	*Anaerococcus*	1	*Anaerococcus*	*vaginalis*	97.24
		2	*Anaerococcus*	*obesiensis*	2.67
		3	*Anaerococcus*	*senegalensis*	0.10
Otu79	*Anaerococcus*	1	*Anaerococcus*	*mediterraneensis*<br>*murdochii*	42.22
		2	*Anaerococcus*	*prevotii*<br>*tetradius*	36.79
		3	*Anaerococcus*	*lactolyticus*	15.56
		4	*Anaerococcus*	*mediterraneensis*<br>*prevotii*<br>*murdochii*<br>*tetradius*	5.43
Otu84	*Peptoniphilus*	1	*Peptoniphilus*	*lacrimalis*	100.00
Otu102	*Ruminococcus*	1	*Ruminococcus*	*bromii*	100.00
Otu105	*Peptoniphilus*	1	*Peptoniphilus*	*grossensis*	47.49
		2	*Peptoniphilus*	*lacydonensis*	46.21

				gorbachii	
		3	*Peptoniphilus*	*harei*	6.13
		4	*Peptoniphilus*	*tyrrelliae*	0.18
Otu106	Uncultured Clostridiales	1	*Casaltella*	*massilensis*	100.00
Otu120	*Bacteroides*	1	*Bacteroides*	*stercoris*	100.00
Otu126	*Parabacteroides*	1	*Parabacteroides*	*merdae*	100.00
Otu158	*Bacteroides*	1	*Bacteroides*	*finegoldii*	37.12
		2	*Bacteroides*	*xylanisolvens*	35.23
		3	*Bacteroides*	*kribbi* *koreensis*	15.15
		4	*Bacteroides*	*ovatus*	12.50
Otu163	*Dorea*	1	*Dorea*	*longicatena*	100.00
Otu207	*Barnesiella*	1	*Barnesiella*	*intestinihominis*	100.00
Otu252	Uncultured Ruminococcaceae	1	*Gemmiger*	*formicilis*	100.00
Otu254	*Lachnospira*	1	*Eubacterium*	*eligens*	100.00
Otu300	*Odoribacter*	1	*Odoribacter*	*splanchnicus*	100.00

Oligotyping was performed on OTUs of interest in an independent validation cohort[15] to delineate their correlation with the taxa of interest in the main study. Biomarkers associated with CDI and non-CDI patients were identified by utilizing LEfSe (LDA > 2.0). CDI: patients with confirmed *C. difficile* infection. Non-CDI: patients with non-*C. difficile* diarrhea or non-diarrheic patients. LDA: Linear discriminant analysis score. LEfSe: Linear discriminant analysis effect size. OTU: Operational taxonomic unit.

Supplementary Table 9: **Validation of identified microbiota-based markers predictive of CDI in an independent patient cohort**

		ANTICIPATE dataset				Validation dataset			
OTU	OTU classification	Patient group	Average relative abundance (%)	LDA	p-value	Patient group	Average relative abundance (%)	LDA	p-value
Otu1	*Enterococcus*	CDI	1.86	4.27	0.0001	CDI	6.02	4.41	0.0037
Otu9	*Finegoldia*	CDI	4.18	4.32	0.0200	Non-CDI	2.82	3.95	0.0009
Otu11	Uncultured Ruminococcaceae	-	0.23	-	-	CDI	1.98	3.97	0.0190
Otu12	*Clostridium XI*	-	0.25	-	-	CDI	1.11	3.95	0.0065
Otu17	*Corynebacterium*	-	0.42	-	-	CDI	0.25	3.55	0.0026
Otu6	*Faecalibacterium*	-	2.07	-	-	CDI	1.73	3.63	0.0137
Otu30	*Blautia*	Non-CDI	2.28	3.83	0.0076	-	0.21	-	-
Otu31	*Ruminococcus2*	Non-CDI	1.32	3.53	0.0059	Non-CDI	2.09	3.70	0.0302
Otu56	*Porphyromonas*	Non-CDI	1.29	3.77	0.0253	-	0.00	-	-
Otu69	*Porphyromonas*	Non-CDI	0.86	3.63	0.0164	-	0.00	-	-
Otu68	*Alistipes*	Non-CDI	0.78	3.35	0.0379	-	0.97	-	-
Otu64	*Ezakiella*	Non-CDI	0.77	3.59	0.0320	Non-CDI	1.20	3.79	0.0190
Otu280	*Bifidobacterium*	Non-CDI	0.73	3.53	0.0411	-	1.02	-	-
Otu2540	*Ruminococcus*	Non-CDI	0.65	3.40	0.0263	-	0.05	-	-
Otu648	*Blautia*	Non-CDI	0.54	3.29	0.0156	-	0.79	-	-
Otu137	Uncultured Lachnospiraceae	Non-CDI	0.42	3.14	0.0487	-	0.06	-	-
Otu106	Uncultured Clostridiales	Non-CDI	0.34	3.18	0.0117	Non-CDI	1.11	4.06	0.0006
Otu974	*Roseburia*	Non-CDI	0.23	3.02	0.0032	-	0.23	-	-
Otu300	*Odoribacter*	Non-CDI	0.20	2.79	0.0496	Non-CDI	0.40	3.64	0.0022
Otu2858	Uncultured Lachnospiraceae	Non-CDI	0.19	2.85	0.0272	-	0.12	-	-
Otu399	Uncultured Ruminococcaceae	Non-CDI	0.14	2.86	0.0214	-	0.02	-	-
Otu294	Uncultured Lachnospiraceae	Non-CDI	0.14	2.79	0.0403	-	0.14	-	-
Otu262	*Butyricoccus*	Non-CDI	0.13	2.84	0.0020	-	0.20	-	-
Otu1827	Uncultured Lachnospiraceae	Non-CDI	0.08	2.54	0.0037	-	0.00	-	-
Otu781	Uncultured Clostridiales	Non-CDI	0.03	2.62	0.0003	-	0.00	-	-
Otu409	*Flavonifractor*	Non-CDI	0.03	2.57	0.0492	-	0.00	-	-
Otu63	*Bacteroides*	-	1.51	-	-	Non-CDI	3.26	3.90	0.0087
Otu37	Uncultured Clostridiales	-	1.92	-	-	Non-CDI	2.55	4.42	0.0001
Otu75	*Anaerococcus*	-	1.08	-	-	Non-CDI	1.95	4.00	0.0002
Otu105	*Peptoniphilus*	-	1.74	-	-	Non-CDI	1.92	4.05	0.0042
Otu79	*Anaerococcus*	-	0.79	-	-	Non-CDI	1.85	4.02	0.0376
Otu14	*Prevotella*	-	1.06	-	-	Non-CDI	1.82	4.16	0.0240
Otu126	*Parabacteroides*	-	0.43	-	-	Non-CDI	1.79	4.23	0.0000

Otu158	*Bacteroides*	-	0.61	-	-	Non-CDI	1.15	3.72	0.0047
Otu28	*Peptostreptococcus*	-	0.62	-	-	Non-CDI	1.07	3.74	0.0277
Otu26	*Akkermansia*	-	1.66	-	-	Non-CDI	1.06	3.77	0.0118
Otu252	Uncultured Ruminococcaceae	-	0.38	-	-	Non-CDI	0.88	3.76	0.0169
Otu84	*Peptoniphilus*	-	0.51	-	-	Non-CDI	0.72	3.70	0.0081
Otu254	*Lachnospira*	-	0.28	-	-	Non-CDI	0.67	3.75	0.0019
Otu16	*Bacteroides*	-	0.72	-	-	Non-CDI	0.57	3.65	0.0012
Otu163	*Dorea*	-	0.41	-	-	Non-CDI	0.52	3.47	0.0080
Otu102	*Ruminococcus*	-	0.26	-	-	Non-CDI	0.52	3.53	0.0022
Otu120	*Bacteroides*	-	0.22	-	-	Non-CDI	0.49	3.71	0.0033
Otu207	*Barnesiella*	-	0.28	-	-	Non-CDI	0.28	3.55	0.0298

Microbiota-based biomarkers identified in the main study were validated by conducting biomarker identification in an independent validation cohort[15]. Potential biomarkers associated with CDI (n = 24) and non-CDI (n = 25) patients were identified in the validation cohort by utilizing LEfSe (LDA > 2.0). OTU9 classified as *Finegoldia* was associated with patients developing CDI (n = 14) when comparing with non-CDI patients (n = 733) in the main study. The non-CDI group in the main study contain patients developing AAD as well as non-diarrheic patients, and further investigation (Supplementary Tables 3 and 5) revealed this OTU to be strongly associated with patients developing AAD at D1. AAD: patients with non-*C. difficile* antibiotic-associated diarrhea. CDI: patients with confirmed *C. difficile* infection. ND: non-diarrheic patients. Non-CDI: patients with non-*C. difficile* diarrhea or non-diarrheic patients. D1: rectal swab sample collected upon study enrollment. LDA: Linear discriminant analysis score. LEfSe: Linear discriminant analysis effect size. OTU: Operational taxonomic unit.

Supplementary Table 10: **Characterization of class-specific longitudinal alterations in microbial composition as a result of broad-spectrum antibiotic treatment**

Antibiotic class	Timepoint	OTU	LDA	p-value	Genus
PBL	D1	Otu105	3.73	0.000	*Peptoniphilus*
		Otu37	3.69	0.000	Uncultured Clostridiales
		Otu94	3.65	0.000	*Streptococcus*
		Otu56	3.63	0.000	*Porphyromonas*
		Otu75	3.63	0.000	*Anaerococcus*
		Otu109	3.61	0.000	*Peptoniphilus*
		Otu69	3.47	0.001	*Porphyromonas*
		Otu79	3.42	0.000	*Anaerococcus*
		Otu62	3.35	0.000	*Streptococcus*
		Otu92	3.29	0.001	*Dialister*
		Otu64	3.23	0.007	*Ezakiella*
		Otu48	3.12	0.002	*Prevotella*
		Otu106	3.12	0.000	Uncultured Clostridiales
		Otu108	3.06	0.000	*Campylobacter*
	D6	Otu1	4.40	0.000	*Enterococcus*
		Otu5	4.10	0.000	*Sphingomonas*
		Otu6	3.96	0.023	*Faecalibacterium*
		Otu1392	3.74	0.000	*Clostridium XlVa*
		Otu61	3.49	0.027	*Parabacteroides*
		Otu1403	3.05	0.000	*Clostridium XlVa*
OBL	D1	Otu2	4.06	0.010	*Escherichia/Shigella*
		Otu30	3.94	0.000	*Blautia*
		Otu8	3.70	0.014	*Streptococcus*
		Otu117	3.67	0.000	*Roseburia*
		Otu43	3.62	0.000	*Collinsella*
		Otu648	3.43	0.038	*Blautia*
		Otu2540	3.37	0.023	*Ruminococcus*
		Otu94	3.36	0.000	*Streptococcus*
		Otu974	3.31	0.000	*Roseburia*
		Otu3	3.18	0.009	*Staphylococcus*
		Otu163	3.17	0.000	*Dorea*
		Otu2858	3.13	0.000	Uncultured Lachnospiraceae
		Otu97	3.11	0.000	*Anaerostipes*
		Otu527	3.10	0.000	*Coprococcus*
		Otu78	3.06	0.005	*Romboutsia*
		Otu137	3.05	0.000	Uncultured Lachnospiraceae
	D6	Otu1	4.79	0.000	*Enterococcus*
		Otu167	3.32	0.000	Uncultured Erysipelotrichaceae

		Otu1392	3.21	0.000	*Clostridium XlVa*
		Otu5	3.08	0.002	*Sphingomonas*
FQN	D1	Otu37	3.99	0.031	Uncultured Clostridiales
		Otu25	3.93	0.049	*Campylobacter*
		Otu2	3.78	0.001	*Escherichia/Shigella*
		Otu105	3.68	0.000	*Peptoniphilus*
		Otu79	3.61	0.000	*Anaerococcus*
		Otu48	3.55	0.000	*Prevotella*
		Otu56	3.41	0.001	*Porphyromonas*
		Otu109	3.41	0.026	*Peptoniphilus*
		Otu94	3.38	0.000	*Streptococcus*
		Otu18	3.35	0.000	*Prevotella*
		Otu69	3.35	0.004	*Porphyromonas*
		Otu68	3.25	0.016	*Alistipes*
		Otu280	3.24	0.012	*Bifidobacterium*
		Otu110	3.20	0.000	*Porphyromonas*
		Otu84	3.15	0.013	*Peptoniphilus*
		Otu96	3.07	0.045	*Alistipes*
		Otu300	3.02	0.000	*Odoribacter*
	D6	Otu5	4.03	0.019	*Sphingomonas*
		Otu3	3.98	0.001	*Staphylococcus*
		Otu10	3.94	0.023	*Prevotella*
		Otu24	3.92	0.046	*Prevotella*
		Otu63	3.83	0.016	*Bacteroides*
		Otu92	3.14	0.039	*Dialister*

The microbiota of patients treated with PBL (n = 194), OBL (n = 133), and FQN (n = 63) was compared at D1 and D6 to identify the class-specific impact of broad-spectrum antibiotic treatment. Distinctly abundant OTUs associated with each timepoint were identified using linear discriminant analysis effect size (LEfSe, LDA > 3.0). D1: rectal swab sample collected upon study enrolment. D6: rectal swab collected approximately six days after initiation and at the end of antibiotic treatment. PBL: penicillin + beta-lactamase inhibitor. OBL: other beta-lactamase antibiotics. FQN: fluoroquinolones. LDA: Linear discriminant analysis score. OTU: Operational taxonomic unit.

Supplementary Table 11. **Primers and overhang adapter sequences utilized in this study**

Primer name	Sequence
341F	5’-CCT ACG GGN GGC WGC AG -3’
Illumina overhang forward	5’-TCG TCG GCA GCG TCA GAT GTG TAT AAG AGA CAG-‘3
802R	5’-GAC TAC HVG GGT ATC TAA TCC -3’
Illumina overhang reverse	5’-GTC TCG TGG GCT CGG AGA TGT GTA TAA GAG ACA G-'3

Supplementary Table 12: **Reference genomes used for mapping of shotgun metagenomic sequences to identify the most abundant species within distinct taxa/OTUs**

Genus	Species	Reference
Agathobaculum	*butyriciproducens*	GCF_003096535.1_ASM309653v1
Agathobaculum	*desmolans*	GCF_000701665.1_ASM70166v1
Alistipes	*finegoldii*	NC_018011.1
Alistipes	*onderdonkii*	NZ_AP019734.1
Alistipes	*timonensis*	GCF_900107675.1_IMG-taxon_2693429854
Bacteroides	*coagulans*	No reference available
Bifidobacterium	*adolescentis*	NZ_CP028341.1
Bifidobacterium	*catenulatum*	GCF_002075855.1_Bbif1899B
Bifidobacterium	*dentium*	NZ_AP012326.1
Bifidobacterium	*faecale*	No reference available
Bifidobacterium	*kashiwanohense*	No reference available
Bifidobacterium	*pseudocatenulatum*	GCF_003952825.1_ASM395282v1
Bifidobacterium	*stercoris*	Synonym to *B. adolescentis*
Blautia	*faecis*	GCF_013302415.1_ASM1330241v1
Blautia	*luti*	GCF_013304735.1_ASM1330473v1
Blautia	*obeum*	NZ_DS264342.1
Blautia	*wexlerae*	NZ_CYZN01000001.1
Butyricoccus	*faecihominis*	GCF_008830385.1_ASM883038v1
Casaltella	*massiliensis*	No reference available
Clostridium	*glycyrrhizinilyticum*	No reference available
Dorea	*formicigenerans*	GCF_000169235.1_ASM16923v1
Enterococcus	*durans*	NZ_CP022930.1
Enterococcus	*faecalis*	NC_004668.1
Enterococcus	*faecium*	NZ_CP039729.1
Enterococcus	*hirae*	NZ_CP023011.2
Enterococcus	*ratti*	GCF_001886195.1_ASM188619v1
Enterococcus	*villorum*	GCF_000407205.1_Ente_vill_ATCC700913_V2
Eubacterium	*eligens*	NC_012778.1
Eubacterium	*hallii*	NZ_LT907978.1
Finegoldia	*magna*	NC_010376.1
Intestinibacillus	*massiliensis*	GCF_900155735.1_ASM90015573v1
Lactonifactor	*longoviformis*	GCF_900129135.1_IMG-taxon_2585428044
Odoribacter	*splanchnicus*	NC_015160.1
Porphyromonas	*asaccharolytica*	NC_015501.1
Porphyromonas	*bennonis*	GCF_000375645.1_ASM37564v1
Porphyromonas	*uenonis*	GCF_000482365.1_ASM48236v1
Roseburia	*inulinivorans*	GCF_000174195.1_ASM17419v1
Ruminococcus	*bromii*	GCF_900101355.1_IMG-taxon_2593339225
Ruminococcus	*faecis*	GCF_001312505.1_ASM131250v1

Ruminococcus	*lactaris*	GCF_000155205.1_ASM15520v1
Ruminococcus	*torques*	GCF_000153925.1_ASM15392v1

Species associated with oligotypes identified after LEfSe analysis when comparing patients developing CDI with all others at baseline (Supplementary Table 6) were utilized to create a manual Kraken 2 database utilizing the genomes in the table as references. Where available, complete genomes were utilized, otherwise an assembly or shotgun metagenomic sequences were utilized.

Supplementary Figures

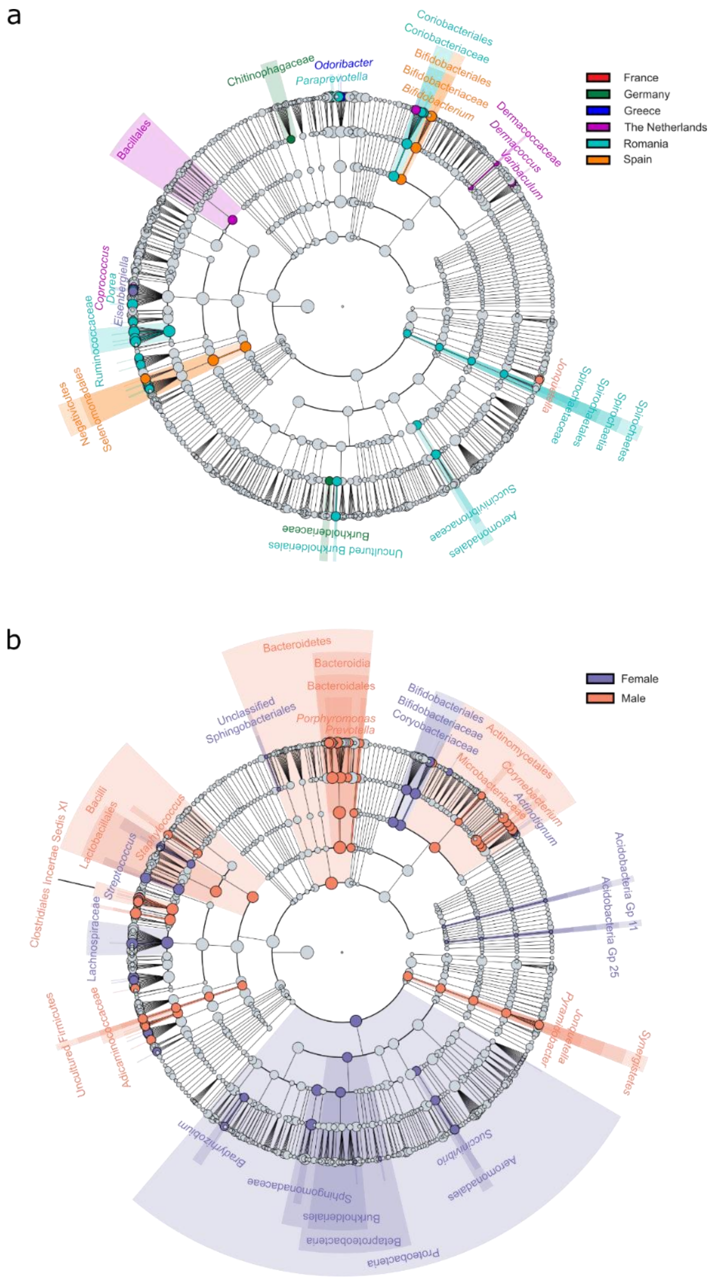

Supplementary Figure 1: **Identification of country- and gender-specific microbial markers of the intestinal microbiota in hospitalized patients.** Differences in the microbiota were assessed at D1 (n = 945) prior to antibiotic treatment in patients stratified by baseline characteristics. **a** Cladogram generated by LEfSe demonstrating distinctly higher abundances (LDA > 2.0) stratified by country of residence of the participating countries France (n = 210, red), Germany (n = 145, green), Greece (n = 85, blue), the Netherlands (n = 14, light purple), Romania (n = 184, light blue), or Spain (n = 307, orange). For more details, see Supplementary Figure 2. **b** Cladogram generated by LEfSe demonstrating distinctly higher abundances (LDA > 2.0) stratified by male (n = 557, salmon) or female (n = 388, purple) gender of the enrolled patients. For more details, see Supplementary Figure 3. M: Male. F:

Female. FR: France. GE: Germany. GR: Greece. NL: the Netherlands. RO: Romania. SP: Spain. LEfSe: Linear discriminant analysis effect size. LDA: Linear discriminant analysis score.

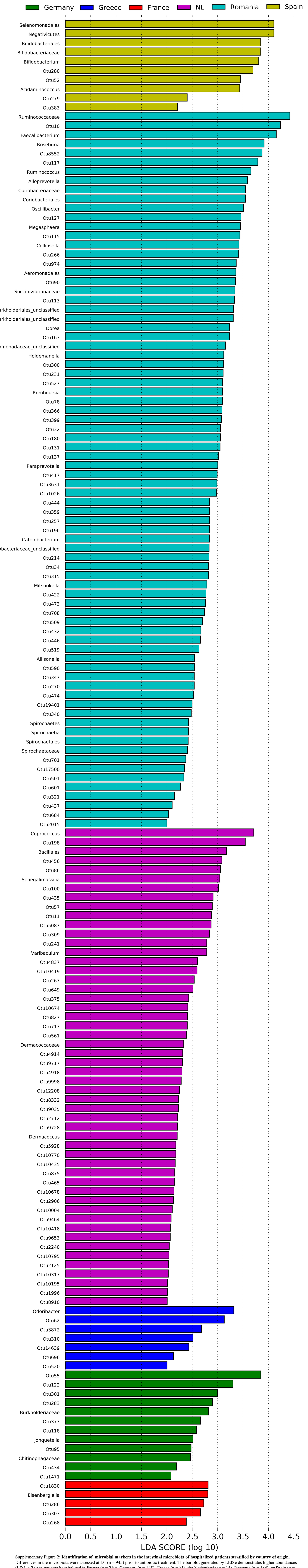

Supplementary Figure 2: **Identification of microbial markers in the intestinal microbiota of hospitalized patients stratified by country of origin.** Differences in the microbiota were assessed at D1 (n = 945) prior to antibiotic treatment. The bar plot generated by LEfSe demonstrates higher abundances (LDA > 2.0) in patients hospitalized in France (n = 210), Germany (n = 145), Greece (n = 85), the Netherlands (n = 14), Romania (n = 184), or Spain (n = 307). For more details, see Supplementary Figure 1a.

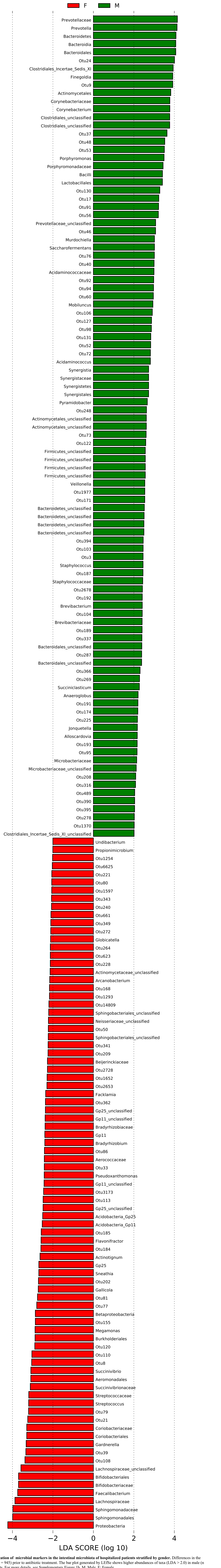

Supplementary Figure 3: **Identification of microbial markers in the intestinal microbiota of hospitalized patients stratified by gender.** Differences in the microbiota were assessed at D1 (n = 945) prior to antibiotic treatment. The bar plot generated by LEfSe shows higher abundances of taxa (LDA > 2.0) in male (n = 557 and female (n = 388) patients. For more details, see Supplementary Figure 1b. M: Male. F: Female.

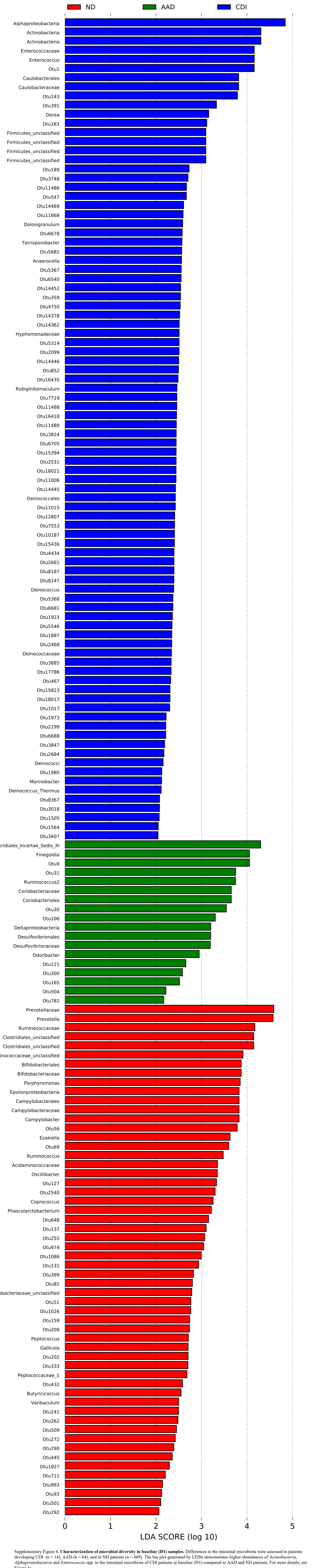

Supplementary Figure 4. **Characterization of microbial diversity in baseline (D1) samples.** Differences in the intestinal microbiota were assessed in patients developing CDI (n = 14), AAD (n = 64), and in ND patients (n = 669). The bar plot generated by LEfSe demonstrates higher abundances of *Actinobacteria*, *Alphaproteobacteria* and *Enterococcus* spp. in the intestinal microbiota of CDI patients at baseline (D1) compared to AAD and ND patients. For more details, see Figure 3c.

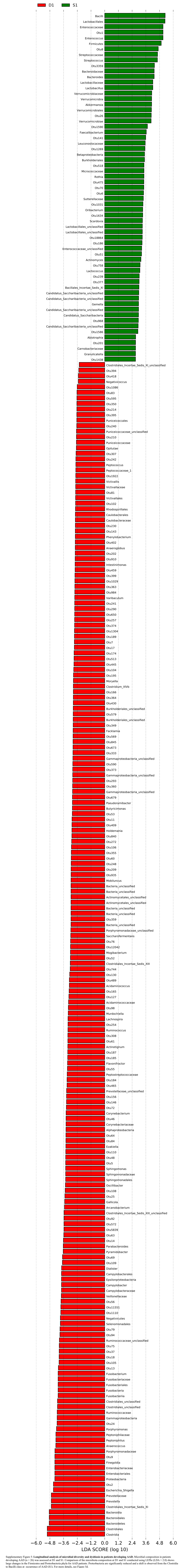

Supplementary Figure 5: **Longitudinal analysis of microbial diversity and dysbiosis in patients developing AAD.** Microbial composition in patients developing AAD (n = 26) was assessed at D1 and S1. Comparison of the microbiota composition at D1 and S1 conducted using LEfSe (LDA > 2.0) shows large changes in the Firmicutes and Proteobacteria phyla for AAD patients. Proteobacteria are significantly reduced and a shift is observed from the Clostridia to Bacilli class at occurrence of AAD (S1). For more details, see Figure 5d.

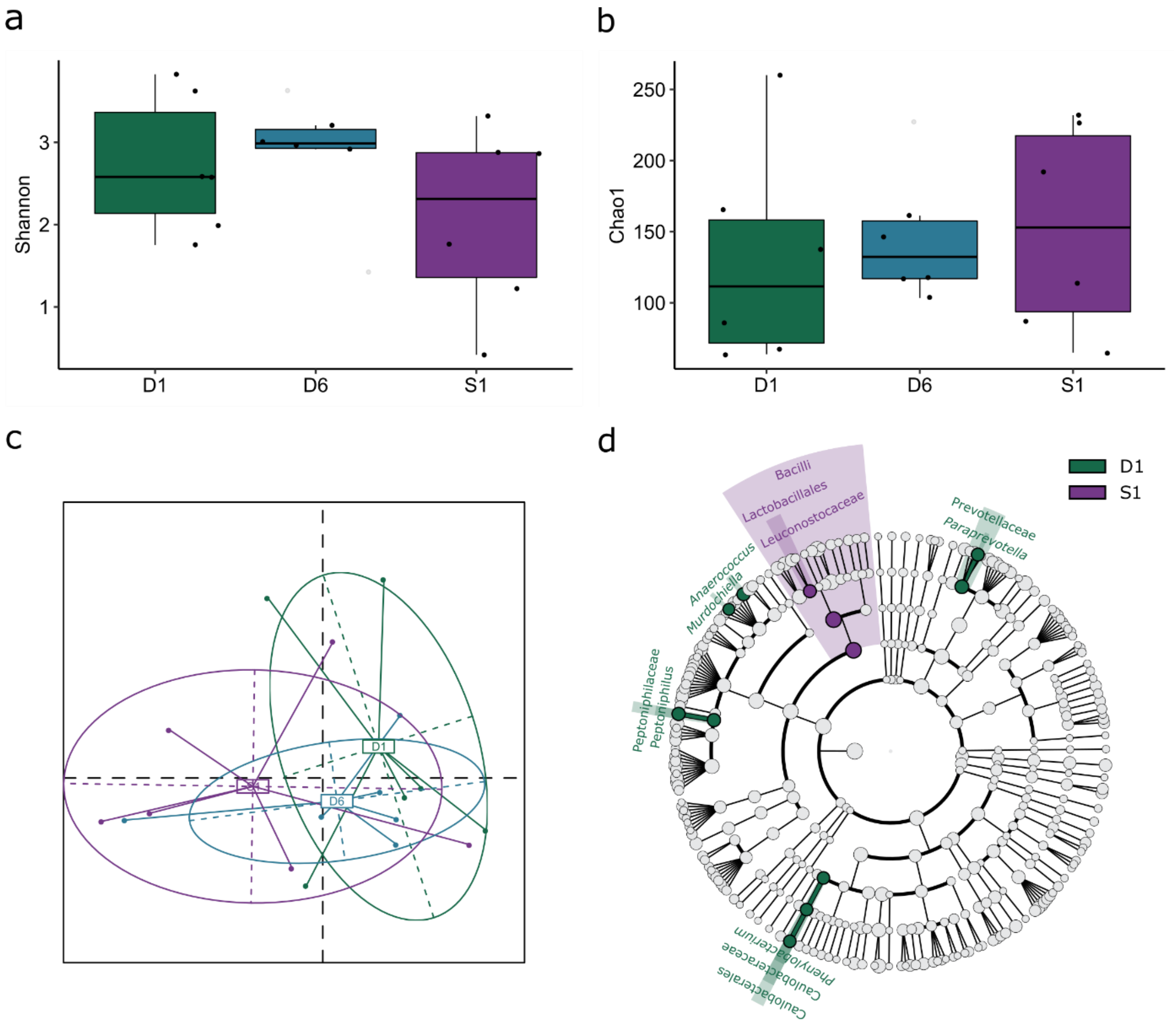

Supplementary Figure 6. **Longitudinal analysis of microbial diversity and dysbiosis in patients developing CDI.** Microbial diversity and composition in patients developing CDI in the study population (n = 6) was assessed at D1 (green), D6 (blue), S1 (purple). Although a gradual decrease was observed in both Shannon and Chao1 diversity in AAD patients (n = 26) between sampling timepoints (Figure 5a-b), no such trend was observed in **a** Shannon or **b** Chao1 diversity in patients developing CDI. **c** Multi-dimensional scaling (MDS) revealed a non-significant trend toward differing microbial communities when comparing composition at different sampling timepoints in CDI patients. **d** Comparison of the D1 and the S1 microbiota conducted using LEfSe (LDA > 2.0) shows few distinct changes for CDI patients, likely due to the small sample size. For more details, see Supplementary Figure 7. Alpha diversity indices were compared using the paired two-sided non-parametric Wilcoxon signed rank test followed by Bonferroni correction of p-values. Box plots indicate median (middle line), 25th, 75th percentile (box), and 5th and 95th percentile (whiskers) as well as outliers (gray single dots)**.** CDI: patients with confirmed *C. difficile* infection.

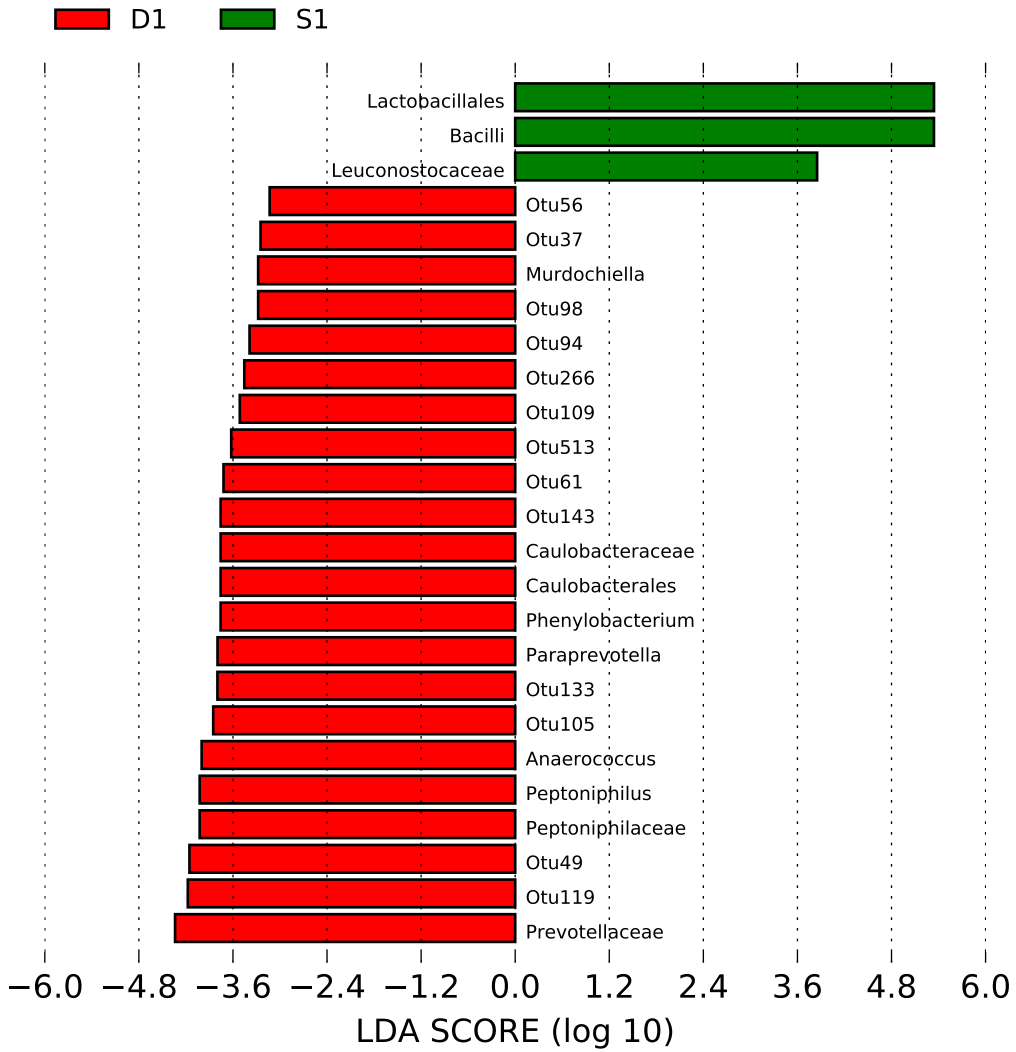

Supplementary Figure 7: **Longitudinal analysis of microbial diversity and dysbiosis in patients developing CDI.** Microbial composition in patients developing CDI (n = 6) was assessed at D1 and S1. Comparison of the D1 and S1 microbiota conducted using LEfSe (LDA > 2.0) shows few distinct changes. For more details, see Supplementary Figure 6d.

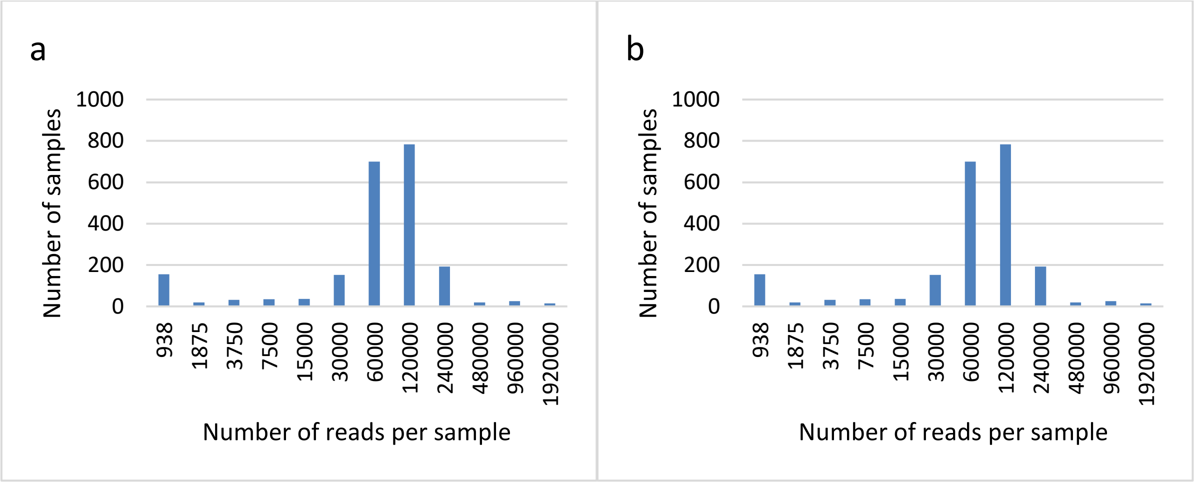

Supplementary Figure 8: **Distribution of raw and processed reads. a** Number of raw reads per sample across all sequenced samples in the study. **b** Number of processed reads per sample across all sequenced samples in the study.

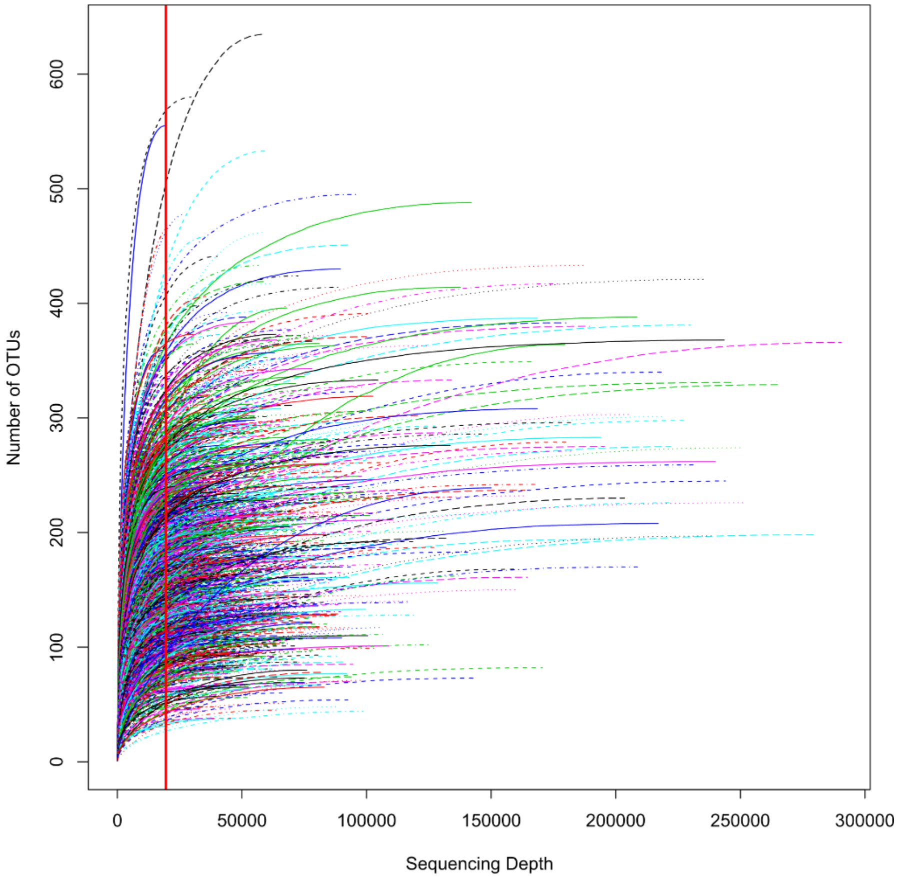

Supplementary Figure 9: **Rarefaction curves for sequenced samples in the study.** Rarefaction curves were calculated in mothur for all sequenced samples in the study (n = 1714). Based on these calculations, a sub-sampling depth of 15,000 was applied on all samples and is indicated in the figure with a red line.

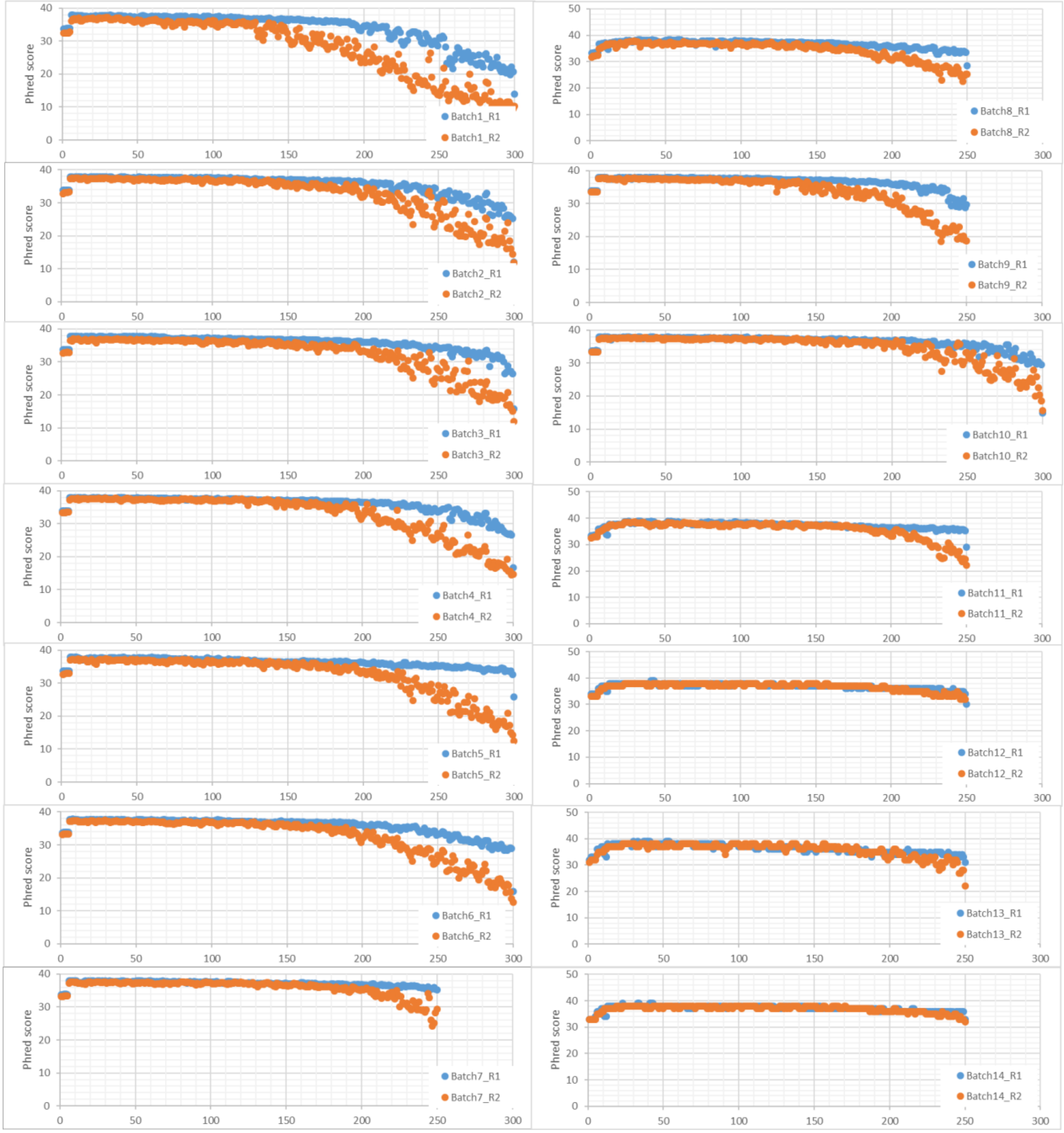

Supplementary Figure 10: **Per-base Phred scores across sequencing batches.**

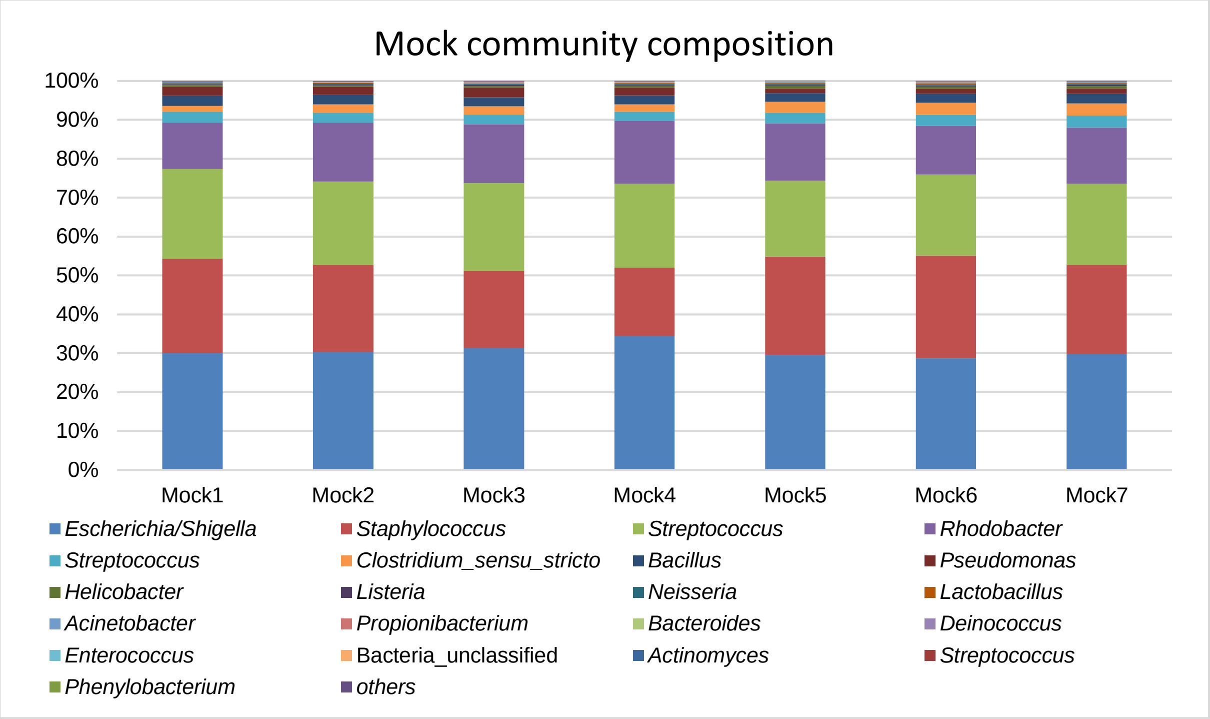

Supplementary Figure 11: **Relative abundance of detected taxa in mock communities.**

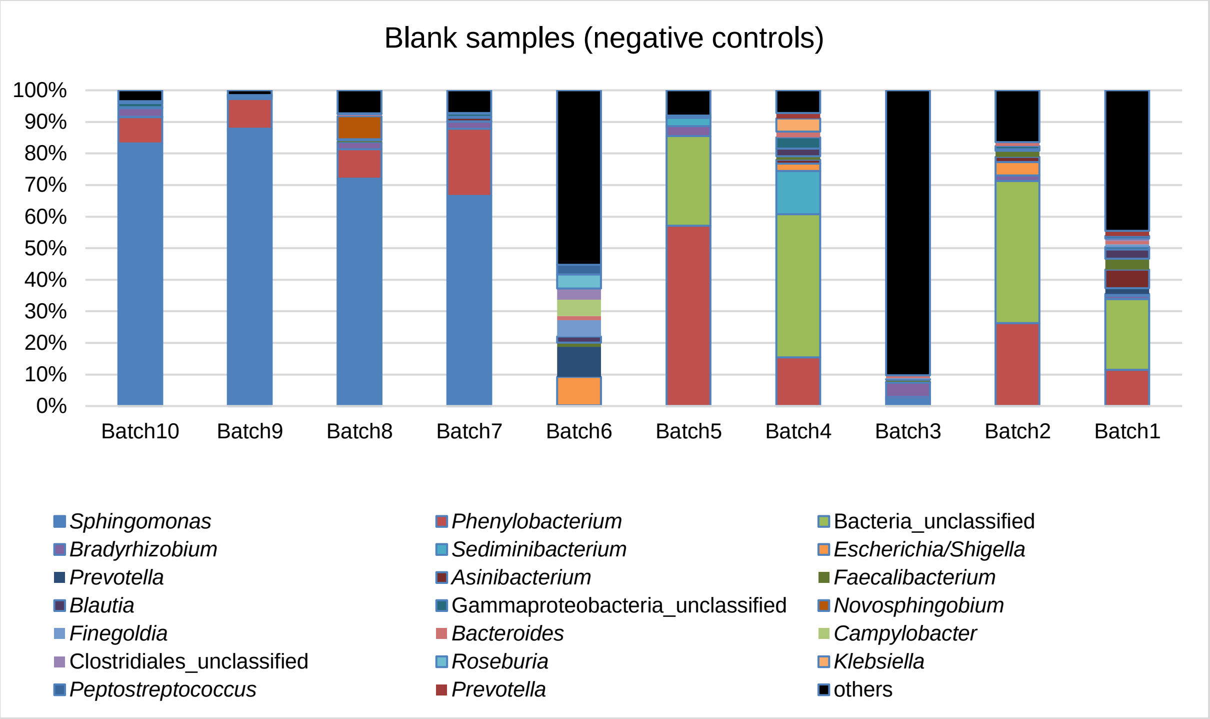

Supplementary Figure 12: **Relative abundances of detected taxa in negative control samples.**

374

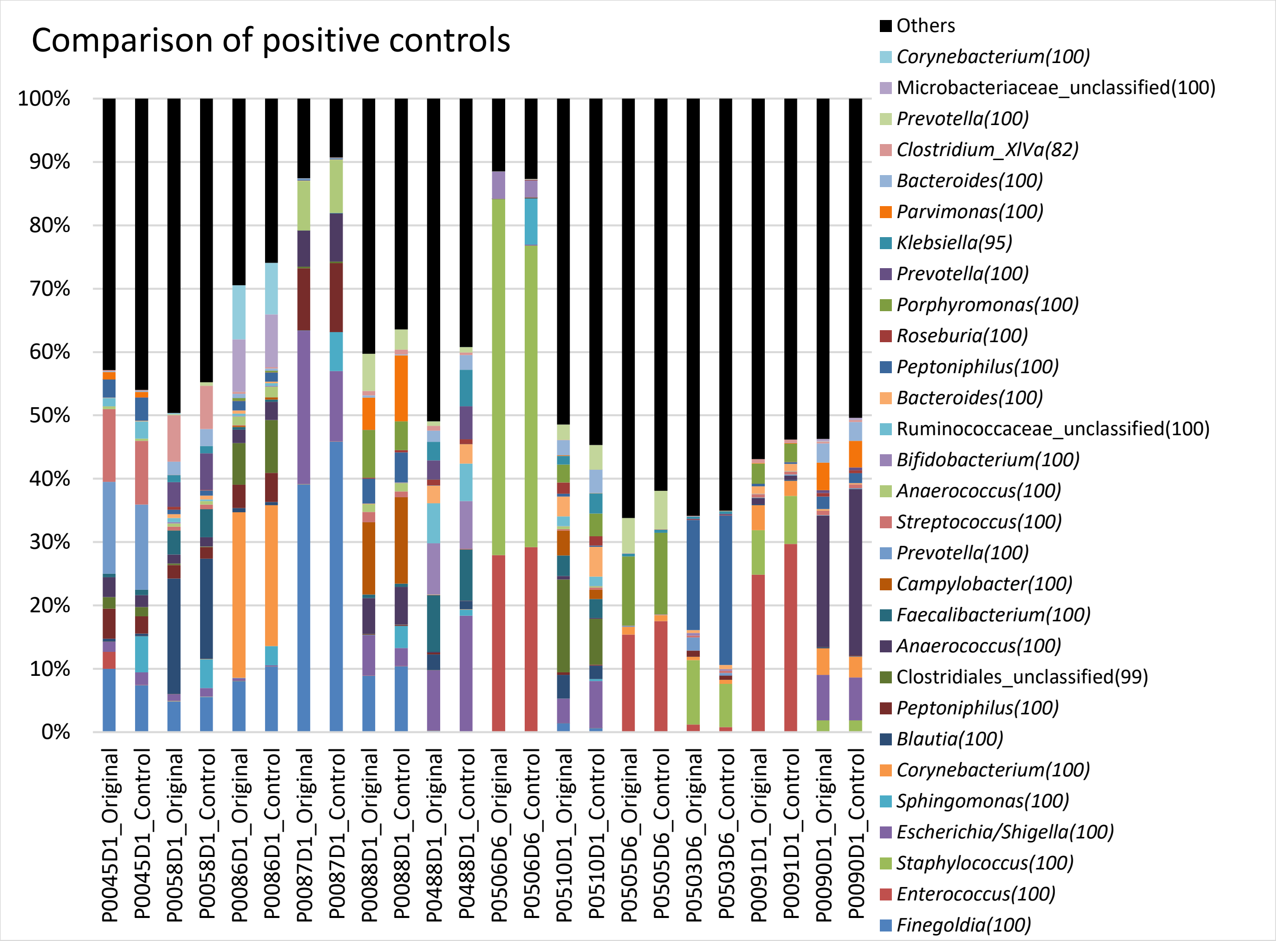

375 Supplementary Figure 13: **Relative abundances of positive patient sample controls.**

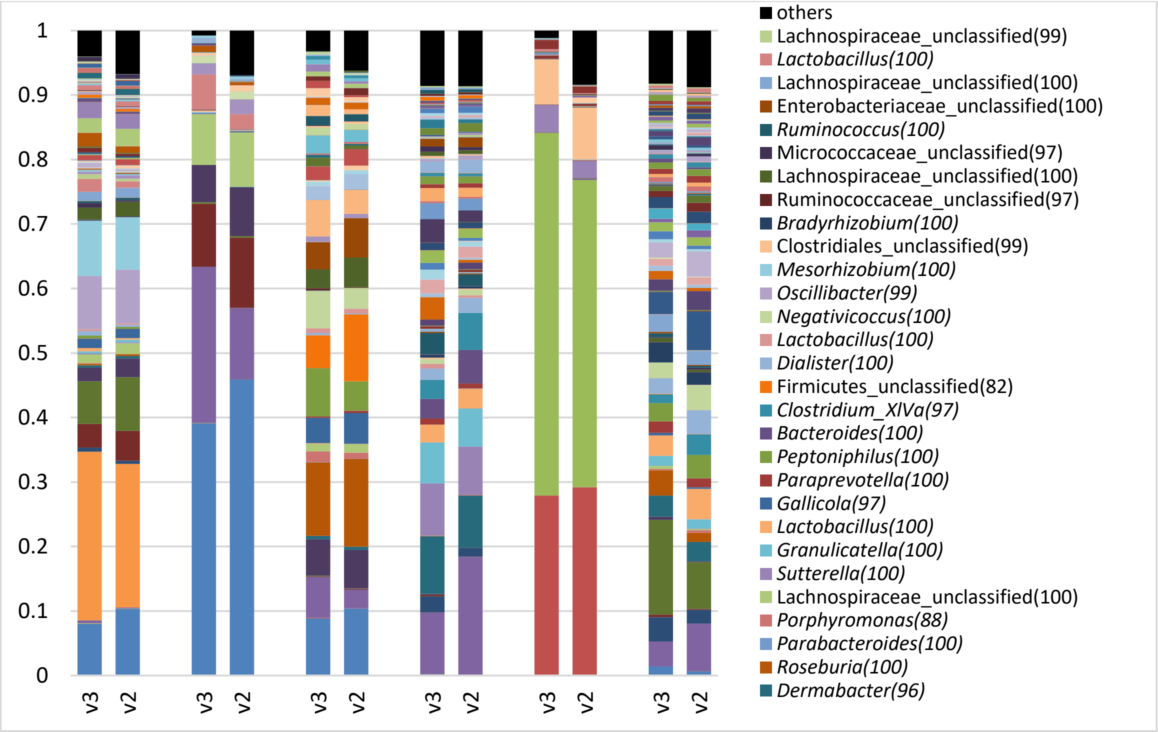

Supplementary Figure 14: **Relative abundances of detected taxa in positive sample controls sequenced with both V2 and V3 chemistry.**

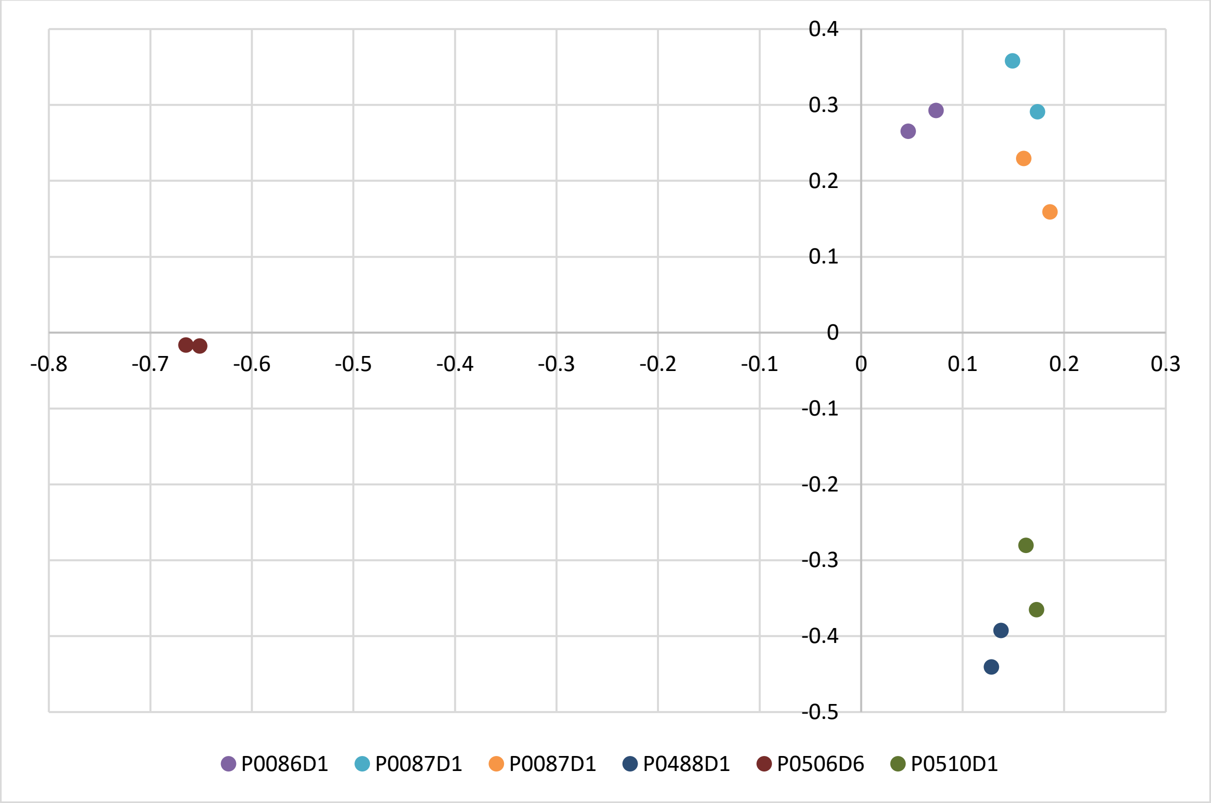

Supplementary Figure 15: **Principle Coordinate Analysis plot visualizing beta diversity of positive sample controls sequenced with both V2 and V3 chemistry.**

Supplementary References

1. Edgar, R. C. Updating the 97% identity threshold for 16S ribosomal RNA OTUs. *Bioinformatics* **34**, 2371–2375 (2018).
2. Smits, W. K., Lyras, D., Lacy, D. B., Wilcox, M. H. & Kuijper, E. J. *Clostridium difficile* infection. *Nat. Rev. Dis. Prim.* **2**, 16020 (2016).
3. Eren, A. M. *et al.* Oligotyping: Differentiating between closely related microbial taxa using 16S rRNA gene data. *Methods Ecol. Evol.* **4**, 1111–1119 (2013).
4. McTeague, M., Finegold, S., Summanen, P., Liu, C. & Song, Y. *Clostridium bartlettii* sp. nov., isolated from human faeces. *Anaerobe* **10**, 179–184 (2004).
5. Lawley, T. D. *et al.* Proteomic and genomic characterization of highly infectious *Clostridium difficile* 630 spores. *J. Bacteriol.* **191**, 5377–5386 (2009).
6. Galperin, M. Y., Brover, V., Tolstoy, I. & Yutin, N. Phylogenomic analysis of the family peptostreptococcaceae (*Clostridium* cluster xi) and proposal for reclassification of *Clostridium litorale* (Fendrich et al. 1991) and *Eubacterium acidaminophilum* (Zindel et al. 1989) as peptoclostridium litorale gen. nov. *Int. J. Syst. Evol. Microbiol.* **66**, 5506–5513 (2016).
7. Callahan, B. J. *et al.* DADA2: High resolution sample inference from Illumina amplicon data. *2Nature Methods* **13**, 581–583 (16AD).
8. Daquigan, N., Seekatz, A. M., Greathouse, K. L., Young, V. B. & White, J. R. High-resolution profiling of the gut microbiome reveals the extent of *Clostridium difficile* burden. *npj Biofilms Microbiomes* **3**, 1–8 (2017).
9. van Werkhoven, C. H. *et al.* Incidence and predictive biomarkers of *Clostridioides difficile* infections in hospitalized patients receiving broad-spectrum antibiotics: a prospective cohort study. *Nat. Commun.* (2021).
10. Salter, S. J. *et al.* Reagent and laboratory contamination can critically impact sequence-based microbiome analyses. *BMC Biol.* **12**, 1–12 (2014).
11. Kennedy, K., Hall, M. W., Lynch, M. D. J., Moreno-Hagelsieb, G. & Neufeld, J. D. Evaluating Bias of Illumina-Based Bacterial 16S rRNA Gene Profiles. *Appl. Environ. Microbiol.* **80**, 5717–5722 (2014).
12. Tremblay, J. *et al.* Primer and platform effects on 16S rRNA tag sequencing. *Front. Microbiol.* **6**, 1–15 (2015).
13. Sze, M. A. & Schloss, P. D. The Impact of DNA Polymerase and Number of Rounds of Amplification in PCR on 16S rRNA Gene Sequence Data. *mSphere* **4**, 1–13 (2019).
14. Kebschull, J. M. & Zador, A. M. Sources of PCR-induced distortions in high-throughput sequencing data sets. *Nucleic Acids Res.* **43**, 1–15 (2015).
15. Vincent, C. *et al.* Reductions in intestinal Clostridiales precede the development of nosocomial *Clostridium difficile* infection. *Microbiome* **1**, 1–11 (2013).
