# Supplementary material for: Microbiota-based markers predictive of development of Clostridioides difficile infection
Source: Nat Commun. 2021 Apr 14;12:2241. doi: 10.1038/s41467-021-22302-0 (PMC8047037; doi:10.1038/s41467-021-22302-0)
Supplement: Supplementary file 2 — Reporting Summary [file 41467_2021_22302_MOESM2_ESM.pdf]

nature research

Corresponding author(s): Surbhi Malhotra-Kumar
Last updated by author(s): Feb 5, 2021

Reporting Summary

Nature Research wishes to improve the reproducibility of the work that we publish. This form provides structure for consistency and transparency in reporting. For further information on Nature Research policies, see our Editorial Policies and the Editorial Policy Checklist.

Statistics

For all statistical analyses, confirm that the following items are present in the figure legend, table legend, main text, or Methods section.

n/a	Confirmed	
☐	☒	The exact sample size (n) for each experimental group/condition, given as a discrete number and unit of measurement
☐	☒	A statement on whether measurements were taken from distinct samples or whether the same sample was measured repeatedly
☐	☒	The statistical test(s) used AND whether they are one- or two-sided<br>*Only common tests should be described solely by name; describe more complex techniques in the Methods section.*
☐	☒	A description of all covariates tested
☐	☒	A description of any assumptions or corrections, such as tests of normality and adjustment for multiple comparisons
☐	☒	A full description of the statistical parameters including central tendency (e.g. means) or other basic estimates (e.g. regression coefficient) AND variation (e.g. standard deviation) or associated estimates of uncertainty (e.g. confidence intervals)
☐	☒	For null hypothesis testing, the test statistic (e.g. F, t, r) with confidence intervals, effect sizes, degrees of freedom and P value noted<br>*Give P values as exact values whenever suitable.*
☒	☐	For Bayesian analysis, information on the choice of priors and Markov chain Monte Carlo settings
☒	☐	For hierarchical and complex designs, identification of the appropriate level for tests and full reporting of outcomes
☒	☐	Estimates of effect sizes (e.g. Cohen's d, Pearson's r), indicating how they were calculated

Our web collection on statistics for biologists contains articles on many of the points above.

Software and code

Policy information about availability of computer code

Data collection	Clinical data was entered electronically into Research Online 2, a Remote Data Capture tool developed by the University Medical Center Utrecht, see https://www.researchonline.info/en-us/ABOUT-US for more information.
Data analysis	Open-source software utilized for 16S rRNA metagenomic analysis: OCToPUS v1.0 (https://github.com/M-Mysara/OCToPUS), mothur v.1.39.1, Illumina Paired-End Denoiser v1.0 (IPED, https://github.com/M-Mysara/IPED/blob/master/README.md), CATCh v1.0 (https://github.com/M-Mysara/CATCh), UPARSE (USEARCH v8.1.186 implementation, https://drive5.com/uparse/), SPAdes v3.5.0, Oligotyping v2.2 (http://merenlab.org/software/oligotyping/), RStudio v.3.5.0, R 3.6.0, LEfSe v1.1.0, Circos (http://circos.ca/), FastQC v0.11.9 (https://www.bioinformatics.babraham.ac.uk/projects/fastqc/), Trim Galore v0.6.4 (https://github.com/FelixKrueger/TrimGalore), Kraken 2 v2.0.9.<br>R packages: Rhea v1.6, ggplot2 v3.3.2.<br>Databases: SILVA v.119 (https://mothur.org/wiki/silva_reference_files/), Ribosomal Database Project (RDP, https://mothur.org/wiki/rdp_reference_files/) v. 16.

For manuscripts utilizing custom algorithms or software that are central to the research but not yet described in published literature, software must be made available to editors and reviewers. We strongly encourage code deposition in a community repository (e.g. GitHub). See the Nature Research guidelines for submitting code & software for further information.

Data

Policy information about availability of data

All manuscripts must include a data availability statement. This statement should provide the following information, where applicable:

- Accession codes, unique identifiers, or web links for publicly available datasets
- A list of figures that have associated raw data
- A description of any restrictions on data availability

Data supporting the findings of this study are available within the paper and its Supplementary Information file. Data underlying Figures 1-5 and Supplementary Figures 1-15 are provided as a Source Data file. 16S rRNA and shotgun metagenomic sequence data generated and analyzed in this study have been deposited in the NCBI Sequence Read Archive with the accession code PRJNA685914. Human reads were identified and removed prior to shotgun metagenomics data upload. All other data generated in this study are available from the corresponding author upon reasonable requests. Raw data from Vincent et. al. utilized as a validation cohort in this study was kindly provided by Prof. Amee Manges (University of British Columbia, Vancouver, Canada). The following public databases were utilized for analysis in this manuscript: SILVA v.119 (https://mothur.org/wiki/silva_reference_files/), Ribosomal Database Project (RDP, https://mothur.org/wiki/rdp_reference_files/) v.16.

Field-specific reporting

Please select the one below that is the best fit for your research. If you are not sure, read the appropriate sections before making your selection.

- [x] Life sciences
- [ ] Behavioural & social sciences
- [ ] Ecological, evolutionary & environmental sciences

For a reference copy of the document with all sections, see nature.com/documents/nr-reporting-summary-flat.pdf

Life sciences study design

All studies must disclose on these points even when the disclosure is negative.

Sample size	No samples were collected in this study. Sample collection was conducted as part of the prospective, observational trial (ClinicalTrial.gov: NCT02896244) described in van Werkhoven et. al. in a back-to-back manuscript (tracing number: NCOMMS-20-23164). A total number of 1007 patients were included in the clinical trial, of which 1002 patients provided (paired) samples. In this 16S-based metagenomics study, fecal samples from 945 patients were available and analyzed at D1 (pre-antibiotic baseline sample) and from 737 patients at D6 (post-antibiotic treatment). In addition, stool samples collected at the occurrence of the first diarrheal episode were available and analyzed for 32 patients.
Data exclusions	In endpoint-specific analyses (CDI versus AAD versus no diarrhea), patients without a recorded clinical endpoint were excluded. For assessment of antibiotic impact, patients not providing paired samples were excluded. For analysis of stools, only patients providing a third (stool) sample at the time of first diarrheal episode were included/analyzed.
Replication	To ensure accuracy and reproducibility of study results, 3 positive controls in the form of biological (patient) samples [to control for batch dependencies], pure C. difficile DNA, and mock communities (HM–783D, https://www.beiresources.org/) were included in all sequencing batches together with negative PCR and DNA extraction controls. Only successfully amplified samples were taken further for sequencing. For complete patient/sample overview, see Figure 1.
Randomization	Observational study, no randomization necessary.
Blinding	Blinding not possible for condition-based analysis conducted in this manuscript.

Reporting for specific materials, systems and methods

We require information from authors about some types of materials, experimental systems and methods used in many studies. Here, indicate whether each material, system or method listed is relevant to your study. If you are not sure if a list item applies to your research, read the appropriate section before selecting a response.

Materials & experimental systems

n/a	Involved in the study	
x		Antibodies
x		Eukaryotic cell lines
x		Palaeontology and archaeology
x		Animals and other organisms
	x	Human research participants
	x	Clinical data
x		Dual use research of concern

Methods

n/a	Involved in the study	
x		ChIP-seq
x		Flow cytometry
x		MRI-based neuroimaging

Human research participants

Policy information about studies involving human research participants

Population characteristics	Total number of patients 945, Age (median [IQR]) 70 [61–79], Male gender 557 (58.9%), Myocardial infarction 78 (8.25%), Congestive heart failure 134 (14.2%), Peripheral vascular disease 143 (15.1%), Cerebrovascular disease 81 (8.57%), COPD 144 (15.2%), Connective tissue disease 53 (5.61%), Peptic ulcer disease 50 (5.29%), Diabetes mellitus 268 (28.4%), Moderate to severe chronic kidney disease 127 (13.4%), Hemiplegia 16 (1.69%), Leukemia 55 (5.82%), Malignant lymphoma 68 (7.20%), Solid tumor 208 (22.0%), Liver disease 88 (9.31%), AIDS 10 (1.06%), Intestinal obstruction 5 (0.53%), Inflammatory bowel disease 14 (1.48%), Other non-specified comorbidities 538 (56.9%), Has history of CDI 14 (1.48%), Developed CDI within study period 14 (1.48%), Developed AAD within study period 64 (6.77%)
Recruitment	No patients were recruited in this study.
Ethics oversight	The study protocol was approved by a central ethics review board in each country and/or the local institutional review boards of each hospital, in accordance with the local regulations. All study participants provided written informed consent for 16S-based metagenomic analysis of fecal samples prior to any study-related activities.<br>Ethical committees:<br>Netherlands: local medical ethics committee approval<br>• UMC Utrecht<br>Germany: local medical ethics committee approvals<br>• UKK Uniklinik Köln<br>• Universitätsklinikum Heidelberg (KLIPPS)<br>• Jena University Hospital<br>• UK-SH (UZL) Universitätsklinikum Schleswig-Holstein, Campus Lübeck<br>• Klinikum der Universität München<br>• Universitätsklinikum Leibzig<br>• University of Aachen<br>• Universitatsklinikum Essen<br>Greece: local medical ethics committee approvals<br>• University Hospital of Heraklion<br>• Laiko General Hospital<br>• Attikon University General Hospital<br>• Evangelismos General Hospital of Athens<br>• Ippokrateio General Hospital of Athens<br>Spain: Central approvals:<br>• Comité Coordinador de Ética de la Investigación Biomédica de Andalucía<br>• Dirección General de Inspección y Ordenación CONSEJERÍA DE SANIDAD Comunidad de Madrid<br>• Local medical ethics committee approvals<br>• Hospital Universitari de Bellvitge<br>• Hospital Universitario 12 de Octubre<br>• Hospital Universitario Gregorio Marañon<br>• Hospital Universitario Ramon y Cajal<br>• Hospital Universitario Virgen Macarena<br>• Hospital Universitari Vall d'Hebrón<br>• Servicio Andaluz de Salud- Reina Sofia University Hospital<br>Romania: Central approval:<br>• Ministry of Health, National Agency for Medicines and Medical Devices<br>• Local medical ethics committee approvals<br>• Infectious and Tropical Diseases Hospital "Dr. Victor Babes"<br>• Clinical Hospital Of Infectious Diseases Of Iasi<br>• The National Institute of Infectious Diseases Matei Bals<br>• Cluj Napoca Infectious disease Clinical Hospital<br>• Oncology Institute Ion Chiricuta<br>France: Central approvals:<br>• ANSM (Agance nationale de sécurité du médicament et des produits de santé)<br>• Comite de protection des personnes du Sud-Ouest et outre-mer IV, Limoges

Note that full information on the approval of the study protocol must also be provided in the manuscript.

Clinical data

Policy information about clinical studies

All manuscripts should comply with the ICMJEguidelines for publication of clinical research and a completedCONSORT checklist must be included with all submissions.

Clinical trial registration	ClinicalTrials.gov: NCT02896244
Study protocol	The study protocol has been provided as a supplemental file.

Data collection	This was a multicenter prospective observational cohort study in 34 hospitals (21 university and 13 non-university hospitals) from France, Germany, Greece, the Netherlands, Romania and Spain. Patients were recruited from September 2016 through October 2017. Data was collected concerning a followup period per participant of 90 days.
Outcomes	Primary outcome: Identification of specific microbial markers or patterns in the intestinal microbiota predisposing to CDI and AAD that could be detected in asymptomatic patients at the time of hospital admission. Secondary outcome: Characterization of changes associated with antibiotic treatment potentially impacting the subsequent risk of CDI and AAD development.<br>These were achieved using 16S rRNA profiling combined with a high-resolution sequence typing approach (oligotyping), and when required, further delineation of species using shotgun metagenomic sequencing.
